# Berberine Enhances Radiosensitivity of Head and Neck Squamous Cell Carcinoma Concurrent with the Inhibition of DNA Repair, Stemness and Tumor Growth

**DOI:** 10.3390/cancers18162690

**Published:** 2026-08-19

**Authors:** Deepali Mishra, Aishwarya Jaiswal, Aleena Sinha, Navneendra Singh, Rana P. Singh

**Affiliations:** 1 Cancer Biology Laboratory, School of Life Sciences, Jawaharlal Nehru University, New Delhi 110067, India; mishradeepali458@gmail.com (D.M.); jaiswalaishwarya93@gmail.com (A.J.); aleenasnh@gmail.com (A.S.); navneendra@gmail.com (N.S.); 2 Gautam Buddha University, Greater Noida 201312, India

**Keywords:** HNSCC, IR, berberine, DNA damage, stemness, apoptosis, syngraft

## Abstract

Background/Objectives: The emergence of radioresistance is a major challenge in cancer treatment. Herein, we evaluated the radiosensitizing effects and associated molecular mechanisms of a small molecule, berberine, in HNSCC. Methods: HNSCC cells were treated with berberine, ionizing radiation (IR), and their combination. Radiosensitivity was assessed using methods for clonogenic survival, proliferation, cell death, apoptosis, cell cycle distribution, DNA damage, and associated mechanisms. Spheroid models were employed to examine tumor growth and stemness-related markers. Therapeutic efficacy was further evaluated using the syngeneic MOC2 tumor syngraft mouse model. Results: The combination of berberine and IR significantly decreased the colony-forming ability, cell proliferation, and survival in UM-SCC-22B and MOC2 cells. Correspondingly, a decrease in the pro-survival signaling, EGFR, ERK1/2, mTOR, and STAT-3 was noted. The combination treatment increased sub-G1 cell population and apoptotic cell death with enhanced DNA damage, which was also associated with downregulation of DNA repair proteins. Spheroids showed disintegration and altered morphology in the combination treatment. This combination also inhibited Wnt/β-catenin signaling and reduced the expression of genes associated with stemness-related pluripotency. Berberine further enhanced DNA damage and reduced stemness in EGFR-knockdown cells. The berberine-IR combination not only inhibited the growth of syngeneic MOC2 tumors but also mitigated the toxicity associated with IR. The *in vitro* findings of reduced expression of stemness and DNA damage repair genes were also observed in tumors. Conclusions: Our findings indicated that berberine enhances the radiation response of HNSCC through perturbation of pro-survival signaling, DDR signaling and reduction of stemness-associated features, thereby enhancing the therapeutic efficacy.

## Simple Summary

Radiotherapy is one of the main treatment options for head and neck cancer, but many tumors gradually become resistant to treatment, reducing its effectiveness and increasing the risk of cancer recurrence. Therefore, identifying compounds that can improve the response of cancer cells to radiation is desired. In this study, we investigated the potential role of a small molecule, berberine, to improve the effectiveness of radiation treatment. We found that berberine increased radiation-induced cancer cell death, reduced cell proliferation and survival, enhanced radiation-induced DNA damage, altered DNA damage response (DDR) pathway proteins, and decreased stemness-associated characteristics, which are often linked to treatment resistance and tumor relapse. In the syngraft mouse model, the combination of berberine and radiation reduced tumor growth. These findings underscore the potential of berberine in enhancing the radiation response and the therapeutic efficacy of radiation against head and neck cancer.

## 1. Introduction

Head and neck cancer (HNC) involves a group of diverse malignancies comprising cancers of the oral cavity, larynx, pharynx, paranasal sinuses, nasal cavity, and salivary glands. It is the seventh most common cancer worldwide, with over 400,000 deaths in 2020 [1], and a 5-year overall survival of approximately 55% [2]. HNC accounts for approximately 30% of all cancers in India, and the disease is mainly linked to smoking, drinking, and the use of tobacco-related products [3]. Prolonged exposure of the upper aerodigestive tract to these carcinogenic substances could lead to the development of dysplastic or premalignant lesions, which can ultimately progress to HNC. In India, it was found that 86% of patients diagnosed with HNC were tobacco users, while 23% were identified as alcohol consumers [4]. HNC accounts for 3% of all cancer cases in the United States, while in India, they represent the most prevalent cancer among men and rank fourth among women. Notably, approximately 90% of HNC are squamous cell carcinoma [5].

Surgery and radiotherapy are presently the standard treatment modalities for the majority of patients with early-stage head and neck squamous cell carcinoma (HNSCC). However, these interventions often lead to considerable morbidity and are linked to an unfavorable prognosis. Additionally, resistance to radiotherapy and chemotherapy, along with the frequent relapse, are prevalent challenges in the management of HNSCC [6]. The five-year survival rate for patients receiving only radiation or chemo-radiation is approximately 50%, while approximately 25% of those treated with radiation experience local relapse [7]. Additionally, these interventions can result in considerable side effects [8]. The aim is to develop a combination with the lowest feasible radiation doses to treat these tumors, thereby achieving optimal therapeutic response while minimizing damage to adjacent healthy tissue.

Resistance to radiation in HNC is associated with modifications in the EGFR signaling pathway and mutations in the p53 gene, both of which play critical roles in the repair of DNA damage caused by radiation [7]. Additional mechanisms contributing to this resistance include Epithelial–Mesenchymal Transition (EMT), deregulation of angiogenic factors, and the presence of cancer stem cells (CSCs). When exposed to ionizing radiation, the DNA damage response (DDR) pathway is activated, enabling cancer cells to recover from radiation-induced DNA damage. Furthermore, cancer stem cells, characterized by their ability to self-renew, demonstrate an inherent capacity for enhanced DNA repair, along with increased invasive and metastatic abilities, anti-apoptotic signaling and reduced pro-apoptotic signaling. Therefore, targeting the pathways involved in DNA damage repair and CSCs presents a promising approach to mitigate radiation resistance in HNC [9].

Numerous plant-derived small molecules have shown potential in the management of radiation resistance by enhancing the treatment response and reducing radiation-induced toxicity. Several studies have demonstrated that berberine, a naturally occurring plant alkaloid, possesses radiosensitizing properties in multiple cancer types. In nasopharyngeal cancer (CNE-2) cells, berberine enhanced radiosensitivity by inducing G0/G1 cell cycle arrest and apoptosis through Sp1 downregulation [10]. Moreover, in MCF-7 spheroids, berberine potentiated the effects of ionizing radiation by reducing cell viability and downregulating the expression of the stemness-related genes, OCT4 and SOX2 [11]. Similarly, berberine increased the sensitivity of the ovarian cancer SKOV-3 cells to radiation [12] and inhibited HIF-1α and VEGF expression, thereby enhancing the radiosensitivity of both ESCC cells and xenografts [13]. Consistently, radiosensitizing effects of berberine have also been reported in prostate cancer and NPC [14,15]. Together, these studies suggest that berberine can enhance radiation responses through diverse mechanisms, including modulation of DNA repair, apoptosis, hypoxia signaling, and cancer stemness. However, whether berberine exerts similar radiosensitizing effects in HNSCC by modulation of DNA damage response pathways and stemness-associated pathways remains largely unexplored. Therefore, in this study, we have investigated the ability of berberine to enhance the radiation response in human HNSCC and mouse oral squamous cell carcinoma (OSCC) cell lines *in vitro* and *in vivo* by modulating DDR signaling and suppressing stemness-related pathways, thereby improving the therapeutic efficacy of radiotherapy.

## 2. Methods

### 2.1. Chemicals and Reagents

Berberine, bromophenol blue, 2′,7′-dichlorofluorescein diacetate (DCFDA), dimethyl sulfoxide (DMSO) molecular grade, ethidium bromide (Et), N-acetyl-L-cysteine (NAC), Ribonuclease A (RNase A), propidium iodide dye (PI), and ethylenediamine tetra acetic acid (EDTA) were procured from Sigma Aldrich (St. Louis, MO, USA). DMEM (Dulbecco’s Modified Eagle Medium, and fetal bovine serum (FBS), were obtained from Gibco (Grand Island, NY, USA). 1% penicillin-streptomycin antibiotic solution was from Himedia (Mumbai, India). Cocktails of protease and phosphatase inhibitors were obtained from Roche Diagnostics (Mannheim, Germany). Polyvinylidene fluoride membrane (PVDF) of 0.45 μm size was purchased from Merck Millipore (Bangalore, India). Primary antibodies against p53, pp53, p21^Cip1^, MDM2, cleaved caspase 3, cleaved PARP, Bcl-2, Bax, p-H2A.X(S139), p-EGFR (Tyr1068), EGFR, p-Akt (Ser473), Akt, p-ERK1/2 (Thr202/Tyr204), ERK1/2, p-STAT3 (Tyr705), STAT3, PTEN, p-mTOR, mTOR, PCNA, Rad51, Oct4, Sox2, Nanog CD44, CD133, Wnt 5a/b, Axin 1, Dvl 2, β-catenin, p-GSK-3β (Ser9), p-Chk1, Chk-1, p-Chk2, Chk2, Ku70, Ku80, p-ATR, ATR, p-ATM, and ATM were all purchased from Cell Signaling Technology (Beverly, MA, USA). Anti-DNA-PK and p27^Kip1^ antibody was purchased from Santa Cruz Biotechnology (Santa Cruz, CA, USA). Anti-β-Actin antibody was from Sigma Aldrich (St. Louis, MO, USA).

### 2.2. Cell Culture

Human oral squamous cancer UM-SCC-22B cell line was authenticated and received from Prof. Shivendra V. Singh’s lab (University of Pittsburgh, School of Medicine, USA), where it was generously gifted by Prof. Daniel E. Johnson (University of California, USA). Mouse oral squamous cancer MOC2 cell line was received as a generous gift from Prof. Sanjay V. Malhotra (Oregon Health & Science University, Portland, USA).

### 2.3. Gamma Irradiation Protocol

Cells were exposed to an ionizing radiation (IR) dose of 2.5 Gy in 1× PBS, subsequently treated with a desired concentration of berberine (1/5 µM). The irradiation was conducted using a standard fac60Co gamma chamber (Model 5000A; BARC, Mumbai, India) at a radiation dose rate of 0.142 Gray per second at the Central Instrument Facility, School of Life Sciences, JNU, New Delhi.

### 2.4. Colony Formation Assay

A total of 600 cells per well were seeded into 6-well culture plates. After 24 h of seeding period, the cells were subjected to 2.5 Gy IR and/or berberine (1 µM for UM-SCC-22B and 5 µM for MOC2), followed by a culture period of 10 to 12 days. The colonies were subsequently fixed using ice-cold methanol for 30 min and stained with a 0.5% solution of crystal violet. Colonies containing more than 50 cells were enumerated using a microscope at a magnification of 100×, as previously reported [16].

### 2.5. Cell Viability Assessment via Trypan Blue Assay

Cells (4 × 10^4^) were seeded in each well of a 12-well culture plate, in triplicate, for each time point. They were then treated with the indicated doses of berberine along with or without IR for 48 h. After the required time point, cells were harvested and resuspended in PBS and mixed with 1% trypan blue dye and counted using a hemocytometer, as previously described [17].

### 2.6. Alkaline Comet Assay

The comet assay was conducted as previously described [18,19]. Cells were harvested, resuspended at a concentration of 5 × 10^4^ cells/mL in 1% low-melting-point agarose, and quickly spread on agarose-coated microscopic slides. Coated slides were kept at 4 °C for 30 min; afterward, slides were immersed in lysis buffer for 2 h and dipped in unwinding buffer for 30 min. The slides underwent horizontal single-cell gel electrophoresis for 20 min at 22-24 V, followed by a 15 min incubation in a neutralization buffer, and staining with EtBr (1 µg/mL). A Nikon TiF fluorescent microscope (Tokyo, Japan) was used to visualize cells in the TRITC channel at 100× magnification. The 2.0 version of Comet score software was used to score the tail DNA percentage.

### 2.7. Cell Cycle Phase Distribution Using Flow Cytometry

Cell cycle analysis was performed as described previously [20]. Briefly, cells were seeded, cultured, and treated with 5 µM of berberine and/or 2.5 Gy radiation for 48 h. Following the treatment, the cells were harvested, stained with an RNase-PI cocktail overnight and analyzed for the phases of cell cycle distribution by flow cytometry (BD FACS Aria Fusion III, BD Biosciences, San Jose, CA, USA).

### 2.8. AO/EtBr Apoptosis Staining

Cells were collected by brief trypsinization after 48 h of treatment, centrifuged, and the pellet was resuspended in 50 μL of PBS. Next, 25 μL of the cell suspension and 2 μL of AO/EtBr solution were mixed in a 1:1 ratio at 10 μg/mL concentration. Immediately, 10 μL of the stained solution was pipetted onto a glass slide and evenly spread using a coverslip. The slides were examined under a Nikon TiF fluorescence microscope (Tokyo, Japan) and cells were counted. The population of apoptotic cells was determined and expressed as a percentage of the total cells counted, as explained earlier [21].

### 2.9. Soft Agar Assay for Colony Formation

The soft agar colony formation assay was employed to assess the anchorage-independent growth. In summary, 2.5 × 10^3^ cells from control, berberine, IR, or their combination treatments were plated in 1.5 mL of semi-solid agar medium (0.37%) within a 6-well cell culture plate. The cells were then incubated at 37 °C in a 5% CO_2_ environment for a duration of 15 days. Following this incubation period, images were captured, and colonies were counted from ten random fields for each condition. 

### 2.10. DCFDA Assay for Reactive Oxygen Species (ROS) Detection

The ROS production was estimated using a DCFDA assay. Cells (1 × 10^4^ per well) were seeded in 96-well culture plates for 24 h, and the cells were pre-treated with 5 mM NAC for 3 h for ROS scavenging. Later on, they were subjected to treatment with 5 µM berberine and 2.5 Gy radiation. H_2_O_2_ (1 mM) served as a positive control. After 48 h, the media were discarded and cells were subsequently incubated with a 10 µM DCFDA solution for 45 min. Finally, the fluorescence was measured using a microplate reader (Thermo Scientific Varioskan Flash Spectral Scanning Multimode Reader, Thermo Fisher Scientific，Waltham, MA, USA), with an excitation and emission filter set at 485 nm and 535 nm, respectively.

### 2.11. Generation of Multicellular Spheroids

Cells/well (300) were seeded in a 96-well plate coated with a thin agarose layer for spheroid formation. Cells were then incubated at 37 °C in a CO_2_ incubator. On day 2, the spheroids were subjected to indicated doses of IR and berberine treatment. Subsequently, on days 4 and 6, 50 μL of fresh berberine-containing medium was added to the treatment wells, while fresh control medium was added to the corresponding control wells, and spheroid growth and morphology were monitored over time, as previously described [22].

### 2.12. Confocal Imaging of Spheroids

Spheroids were stained with cocktail dye Calcein-AM, Hoechst-33342, and Ethidium bromide (EtBr). For staining, spheroids were incubated with 25 µM of Calcein-AM (live, green) and 50 µM of EtBr (dead, red) for 30 min in a CO_2_ incubator and counterstained with 20 µg/mL Hoechst-33342 (DNA, blue). The spheroid images were captured at 100× and 200× magnifications using Nikon A1R HD confocal microscopy (Tokyo, Japan).

### 2.13. Western Blot Analysis

Western blotting was performed following the previously established protocol [18]. Overall, cells were collected after the desired dose of berberine and/ or radiation. At the end of the treatment period, cells were processed for whole-cell lysate preparation. Protein concentration was estimated by Bradford assay, and proteins were separated on a 10–15% sodium dodecyl sulfate-polyacrylamide gel (SDS-PAGE) and transferred onto PVDF membranes and blocked with 3% BSA/skim milk. Membranes were incubated overnight with a specific primary antibody, followed by incubation with the HRP-conjugated secondary antibody. β-actin was used as a loading control. ImageJ software version 1.54g was used for fold change quantification of the band intensity.

### 2.14. EGFR Knockdown in UM-SCC-22B Cells

The receptor tyrosine kinase, EGFR, was knocked down in UM-SCC-22B cells using a short hairpin RNA-based lentiviral system and the pLKO.1 cloning vector. Lentiviral particles were produced utilizing helper plasmids (psPAX2 and pMD2.G), as described earlier [23]. For knockdown, 5 × 10^4^ cells were seeded in a 6-well plate. After 24 h of seeding, polybrene and shEGFR lentiviral particles were added to the knockdown group and polybrene and pLKO.1 vector were added to the control group. After 12 h, the media was changed to complete medium, which, after 48 h, was replaced by puromycin selection medium for the selection of EGFR knockdown cells.

### 2.15. In Vivo Tumor Syngraft Study

The animal experiment was approved by the Institutional Animal Ethics Committee (IAEC) of the Jawaharlal Nehru University (JNU), New Delhi, India. Six animals were included in each experimental group. Mice aged 6 weeks, weighing 20–25 g, of both sexes, were used for the study. Mice were housed at the Central Laboratory Animal Resources, Jawaharlal Nehru University, New Delhi, India, and were maintained under standard conditions. The sample size was determined based on an a priori power analysis using a significance level (α) of 0.05 and a statistical power (1-β) of 0.80. MOC2 cells (1 × 10^5^) mixed with Matrigel were subcutaneously inoculated into the right flank of the C57BL/6 mice. After the implantation, tumor growth in mice was monitored regularly, and once the tumor reached a volume of approximately 150 mm^3^, the mice were randomized into six groups and respective treatments were given. IR at 2.5 Gy/dose was given twice weekly to the IR groups until a cumulative dose of 15 Gy was reached. Berberine was given in 0.9% saline to the mice via oral gavage and continued for 6 days/week. Mice in Group I (vehicle control) received 0.2 mL of 0.9% saline, while Group II mice received a low dose of berberine (7.5 mg/kg); Group III mice were treated with a high dose of berberine (15 mg/kg); Group IV mice were treated with IR alone (15 Gy); Group V mice were treated with IR and low dose of berberine; and Group VI mice were treated with IR and high dose of berberine. Mice were maintained under standard conditions with a 12 h light and 12 h dark cycle, controlled temperature, and humidity. Animals were monitored every alternate day for humane endpoints. Tumor diameter was measured twice weekly with a vernier caliper, and on day 35, mice were euthanized and tumors were dissected out, weighed, and subsequently apportioned into two sections: one used for hematoxylin and eosin staining and immunohistochemistry, and the other for Western blot analysis. Tumor volume measurements and tissue analyses were performed group-wise in an unblinded manner.

### 2.16. Hematoxylin and Eosin Staining

Tissues were fixed in formalin before being embedded in paraffin to prepare 5 µm tissue sections using a microtome (Thermo Scientific, Microm HM 325, Waltham, MA, USA) and placed on microscopic slides. The sections were deparaffinized in xylene and stained with hematoxylin and eosin, and slides were mounted in DPX as previously described [24,25]. Images were captured at 200× magnification with an Olympus microscope (Tokyo, Japan).

### 2.17. Western Blot Analysis of Tumors

For Western blotting, 50 mg of tumor tissue from each tumor was weighed and kept in the Lysis buffer for 2 h on ice. Afterward, the tissue sample was homogenized, centrifuged, and the supernatant was collected. The rest of the steps were followed as mentioned above in the Western blot analysis section.

### 2.18. Immunohistochemical Analysis of Tumors

Tissue sections were deparaffinized in xylene, rehydrated in ethanol, and rinsed with water. The antigen retrieval approach was adopted to expose the epitopes. Further sections were blocked, incubated with specific primary antibodies and then with biotinylated secondary antibodies, followed by 3,3-diaminobenzidine (DAB) staining and lastly counterstained with hematoxylin. Images were captured at 400× magnification with an Olympus microscope (Tokyo, Japan). In order to determine the proportion of Ki-67 positive, brown-stained cells were counted among all cells in five randomly selected fields, as previously explained [24,25]. Densitometry analysis of IHC sections was performed using ImageJ software version 1.54g to quantify cleaved-PARP-positive cells. 

### 2.19. Computational Analysis of H&E-Stained Tumor Section

H&E-stained images of MOC2 tumors treated with berberine and/or IR were analyzed using the Tissue Image Analytics Toolbox (TIAToolbox) with the pretrained HoVer-Net nuclear segmentation and PanNuke classification model [26,27] to segment individual nuclei and classify cells into neoplastic (class 0), inflammatory (class 1), connective tissue (class 2), dead (class 3), and epithelial (class 4) categories [28]. Cell-level annotation files generated by HoVer-Net were exported as CSV files, and the number of cells belonging to each classification category was quantified for each sample. The percentage distribution of each cellular population was calculated based on the total number of detected cells. The obtained cellular composition profiles were compared among experimental groups and visualized using stacked bar graphs.

### 2.20. Densitometric and Statistical Analyses

Western blot films developed with ECL (Merck, MA, USA) were scanned for the bands, and their mean density was analyzed with ImageJ s. Fold changes were calculated after normalization with their respective loading controls (β-actin) and compared with respective controls. The experiments were independently repeated three times and the blots shown in the manuscript are representative of these independent experiments. The densitometric quantification after normalization is derived from the representative blot presented. A statistical analysis of the data was performed using GraphPad Prism software version 8.0.2. The Student’s *t*-test was employed to assess the significance of differences between two groups, with a *p*-value of less than 0.05 deemed significant. For multiple comparisons, ANOVA was performed, followed by the Bonferroni post-test. To compare tumor volume and for the densitometric quantification of Western blot data of syngraft, the statistical analysis was done using paired Student’s *t*-test.

## 3. Results

### 3.1. Berberine Enhances the Radiation Response of Human HNSCC and Mouse OSCC Cells and Inhibits Pro-Survival Signaling Pathways

**Figure 1:**
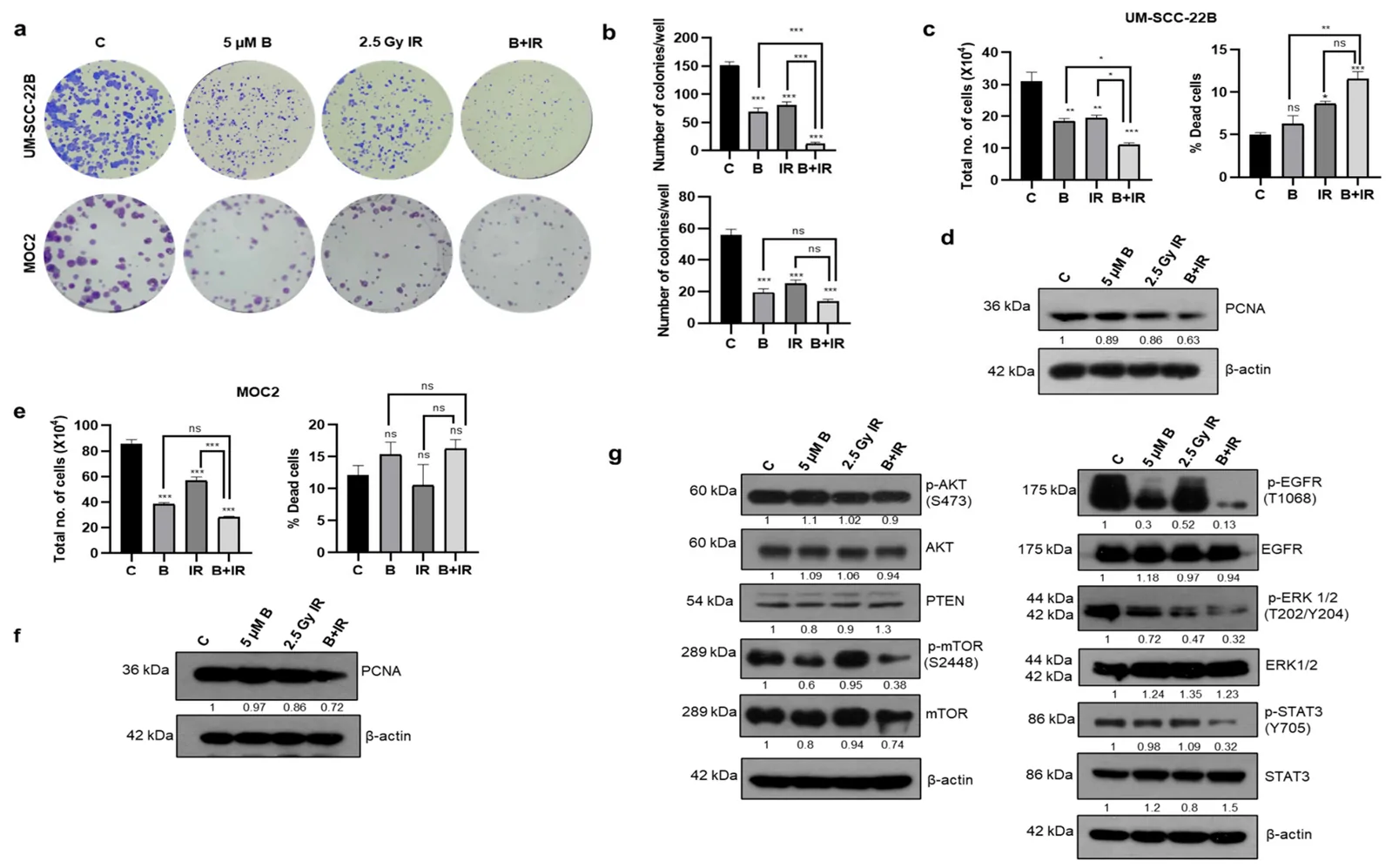
Berberine enhances the response to radiation of human HNSCC and mouse OSCC cells, inhibits cell proliferation, and mitigates pro-survival signaling. (**a**) UM-SCC-22B and MOC2 cells (600/well) were subjected to treatment with 1 and 5 µM berberine, respectively, in combination with 2.5 Gy of radiation, followed by a clonogenic assay performed after a period of 12 days. (**b**) Bar graph depicting the number of colonies. (**c**,**e**) UM-SCC-22B and MOC2 cells (4 × 10^4^ cells/well) seeded in a 12-well plate, and treatments of 5 µM berberine and 2.5 Gy IR were given alone or in combination. After 48 h, cells were harvested, and a trypan blue assay was performed as mentioned in the methods. (**d**,**f**) Western blotting showing the expression of PCNA protein in 5 µM berberine and/or 2.5 Gy IR conditions. (**g**) Western blots depicting the expression of p-EGFR, EGFR, p-ERK1/2, ERK1/2, pSTAT3, STAT3, p-mTOR, mTOR, p-AKT, AKT, and PTEN. β-actin was used as a loading control. The cell growth data represent the mean ± SEM of triplicate samples for each treatment. The experiment was independently repeated three times, with similar results. *, *p* < 0.05; **, *p* < 0.01; ***, *p* < 0.001; ns, non-significant. The *p*-value is computed by comparing each treatment to the control group. C, Control; B, Berberine; IR, Ionizing radiation; B + IR, berberine in combination with radiation.

To assess the radiosensitizing potential of berberine, a clonogenic assay was performed. UM-SCC-22B (human HNSCC) and MOC2 (mouse OSCC) cells were treated with 1 µM and 5 µM berberine, respectively, and/or 2.5 Gy ionizing radiation (IR). Berberine and IR treatment alone resulted in a decrease in colony formation by 54% (*p* < 0.001) and 46% (*p* < 0.001) in UM-SCC-22B cells, and 65% (*p* < 0.001) and 55% (*p* < 0.001) in MOC2 cells; however, the combination treatment led to 92% (*p* < 0.001) and 75% (*p* < 0.001) decrease in UM-SCC-22B and MOC2 cells, respectively, as compared to control (Figure 1a,b). Trypan blue dye exclusion assay was performed to study the combinatorial effects of berberine and IR on cell proliferation using concentrations of berberine and radiation doses based on the initial dose optimization study (Figure S1). Post 48 h treatment, 5 µM berberine and 2.5 Gy IR alone caused a decrease in the total number of cells by 40% (*p* < 0.01) and 37% (*p* < 0.01) in UM-SCC-22B cells, and 55% (*p* < 0.001) and 33% (*p* < 0.001) in MOC2 cells. However, in the combination treatment, the decrease was 64% (*p* < 0.0001) and 67% (*p* < 0.001) in UM-SCC-22B and MOC2 cells, respectively (Figure 1c,e). The expression of PCNA, a marker associated with cell proliferation, was decreased in both cell types (Figure 1d,f and Figure S4: Uncropped Western blot images), highlighting the decrease in cell proliferation following treatments with berberine and IR.

Given the observed decrease in cell proliferation, we subsequently evaluated the impact of berberine and IR on pro-survival signaling molecules, phosphorylated and total forms of EGFR (Tyr1068), AKT(Ser473), ERK1/2 (Thr202/Tyr204), STAT3 (Tyr705), and mTOR under berberine and IR treatment conditions, wherein the combination treatment strongly diminished the EGFR-ERK1/2-mTOR signaling. The levels of phosphorylated STAT3 were decreased, while the total protein levels remained relatively unaltered. In contrast, phosphorylated AKT exhibited only a minor reduction, which corresponded to a slight elevation in its inhibitor, PTEN (Figure 1g, Figures S5 and S6: Uncropped Western blot images). Collectively, these findings suggest that the combination of berberine and IR effectively inhibited cell proliferation and the related pro-survival signaling pathways in both human HNSCC and murine OSCC cells.

### 3.2. Berberine and IR Increase the Sub-G1 Population with Enhanced Apoptosis

**Figure 2:**
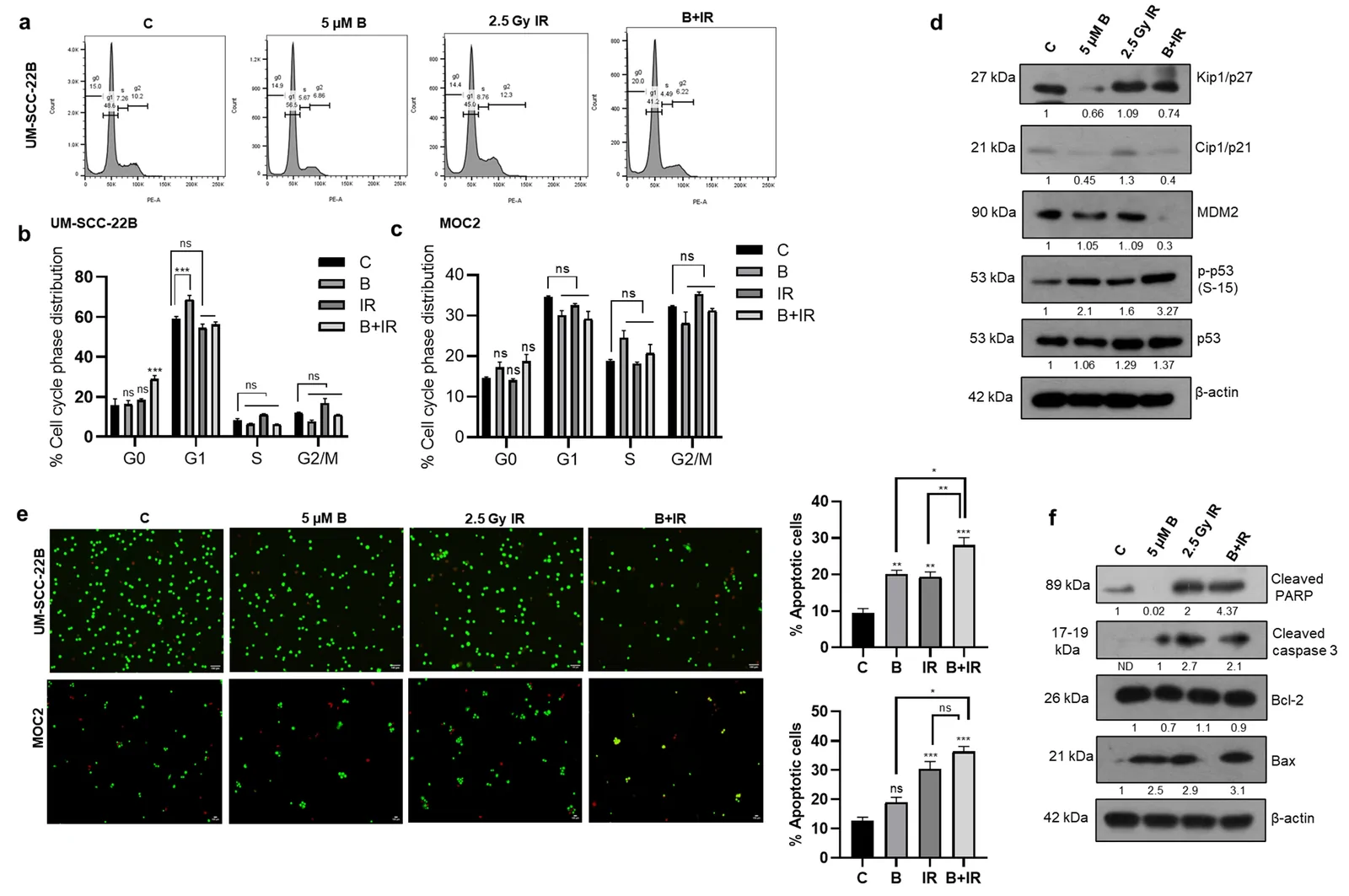
Berberine in combination with radiation induces a sub-G1 population and apoptosis in human HNSCC and mouse OSCC cells. UM-SCC-22B and MOC2 cell lines were cultured at a density of 4 × 10^4^ cells/well in a 12-well plate and subsequently subjected to treatment with berberine and/or ionizing radiation (IR). After a 48 h treatment period, the cells were collected and prepared for cell cycle analysis utilizing flow cytometry. (**a**) Histogram depicting cell cycle distribution at various phases. (**b**,**c**) Quantitative demonstration as a percentage distribution of cells in UM-SCC-22B and MOC2 cells. (**d**) UM-SCC-22B cells were given treatment with berberine and/or radiation, followed by the preparation of total cell lysates as outlined in Section 2. Subsequent analyses included SDS-PAGE and Western blotting to assess cell cycle regulators. To ensure consistent protein loading, the membranes were subsequently stripped and re-probed with a β-actin antibody. (**e**). Cells were treated with berberine and/or radiation, and AO/EtBr assay was performed for the apoptotic cell population as detailed in Section 2. Green-stained cells represent viable cells, yellow-orange cells indicate early apoptotic cells, and orange-stained cells represent late apoptotic cells. Bar graphs illustrating the percent apoptotic cells in UM-SCC-22B and MOC2 cells. (**f**) Western blotting to analyze the expression of key proteins involved in apoptosis. The data represent mean ± SEM of triplicate samples of each treatment. *, *p* < 0.05; **, *p* < 0.01; ***, *p* < 0.001; ns, non-significant. The experiments were independently repeated three times, showing similar results.

Next, we assessed the effects of berberine and IR on the cell cycle phase distribution through flow cytometry and found that berberine alone increases the G1 population from 60% in the control to 67% (*p* = ns) in UM-SCC-22B cells. IR treatment resulted in an increase in the G2/M cell population from 12% in the control cells to 15% in the IR-treated cells. In the combination treatment involving berberine and IR, a substantial increase in sub-G1 cell populations was observed from 18% in the control group to 28% (*p* < 0.001). The increase in sub-G1 cell population in UM-SCC-22B cells was at the expense of a decrease in G1, S, and G2/M phases of the cell cycle (Figure 2a,b). However, in MOC2 cells, the combination treatment led to an increase in the sub-G1 population from 14% in the control to 19% (*p* = ns), which was attributed to a decrease in the G1 and G2/M phases of the cell cycle (Figure 2c). A decrease in the expression of cyclin-dependent kinase inhibitors (CDKI), p21^Cip1^ and p27^Kip1^, was observed with increased levels of phosphorylated and total p53, which is a known mediator of DNA damage and cellular stress, alongside a decrease in the expression level of the p53 inhibitor, MDM2 (Figure 2d and Figure S7: Uncropped Western blot images).

Increased sub-G1 cells are indicative of the presence of apoptosis, which was then confirmed by acridine orange/ ethidium bromide (AO/EtBr) staining. Treatment with berberine alone led to an increase in apoptotic cells by 20% (*p* < 0.01) and by 19% (*p* < 0.01) after IR exposure, as compared to 9% in the control group of UM-SCC-22B cells. An increase in apoptotic cells from 13% in the control group to 19% (*p* = ns) after berberine treatment and to 30% (*p* < 0.001) after IR treatment was observed in MOC2 cells. However, the combination treatment increased the percentage of apoptotic cells to 28% (*p* < 0.001) and 36% (*p* < 0.001) in UM-SCC-22B and MOC2 cells, respectively (Figure 2e). The key molecular markers related to apoptosis, namely cleaved caspase-3 and cleaved PARP, were increased in the combination treatment. Furthermore, the expression level of Bax, a pro-apoptotic protein, was elevated, while that of Bcl2, an anti-apoptotic protein, was reduced, which corresponded to the increased apoptosis as observed in these cells (Figure 2f and Figure S8: Uncropped Western blot images). Further, we investigated the level of reactive oxygen species (ROS) to elucidate its potential role in apoptosis in human HNSCC cells and noted the increased production of ROS in cells in both berberine and IR treatments (Figure S2a).

### 3.3. Berberine and IR Induce DNA Damage in Human HNSCC and Mouse OSCC Cells

**Figure 3:**
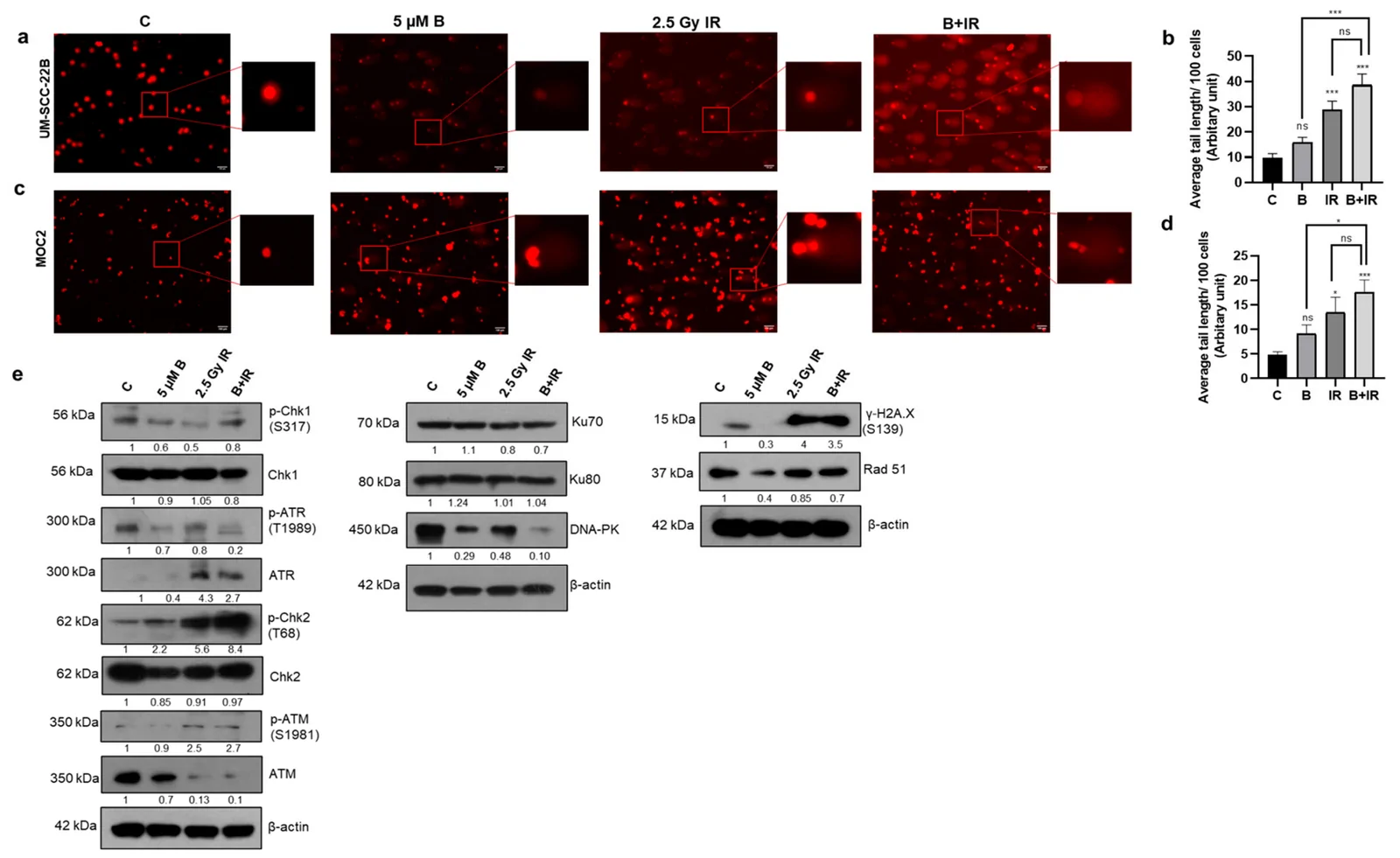
Berberine in combination with radiation induces DNA damage in human HNSCC and mice OSCC cells. (**a**,**c**) A total of 4 × 10^4^ cells per well were cultured in a 12-well plate, and a comet assay to assess DNA damage was conducted on UM-SCC-22B and MOC2 cells at the 48 h duration following treatment with berberine and ionizing radiation, as detailed in Section 2. (**b**) A bar graph representing comet tail length in UM-SCC-22B cells. (**d**) A bar graph representing comet tail length in MOC2 cells. (**e**) Western blot analysis of key DNA damage signaling molecules activated in response to IR and/or berberine. Comet tail length was calculated for 100 cells for each group and statistical analysis was performed. *, *p* < 0.05; ***, *p* < 0.001; ns, non-significant. All experiments were independently performed three times with similar results.

IR can induce damage to DNA, which was confirmed by the alkaline comet assay (Figure 3a,c). Berberine and IR alone caused an increase in comet tail length to 16 a.u. (*p* = ns) and 29 a.u. (*p* < 0.001) as compared to 10 a.u. in the control in UM-SCC-22B cells and an increase to 9 a.u. (*p* = ns) and 14 a.u. (*p* < 0.05) in MOC2 cells, as compared to 5 a.u. in the control. The combination treatment led to an increase in comet tail length to 39 a.u. (*p* < 0.001) and 18 a.u. (*p* < 0.001) in UM-SCC-22B and MOC2 cells, respectively (Figure 3c,d). We also observed a marked increase in γ-H2A.X (Ser139) levels, a known marker of DNA damage, following berberine and radiation treatment, indicating induction of DNA damage, corroborating an increased comet tail length in these conditions (Figure 3e and Figures S9–S11: Uncropped Western blot images).

We then checked the expression of DDR genes in UM-SCC-22B cells and found that the expression of phosphorylated ATR and its downstream effector Chk1, which is usually linked to the response to single-strand DNA damage, was decreased. The phosphorylated ATM and Chk2, which are activated in response to the double-strand breaks (DSBs), were increased, indicating that DSBs were more frequent upon the combination treatment. Also, DNA-PK and Rad51, a known Non-Homologous End Joining (NHEJ) pathway protein and Homologous Recombination (HR) repair protein, were reduced (Figure 3e), indicating the impairment of DNA repair pathway proteins upon treatment with berberine and IR.

### 3.4. Berberine and IR Inhibit Stemness-Associated Properties in Cancer Cells

**Figure 4:**
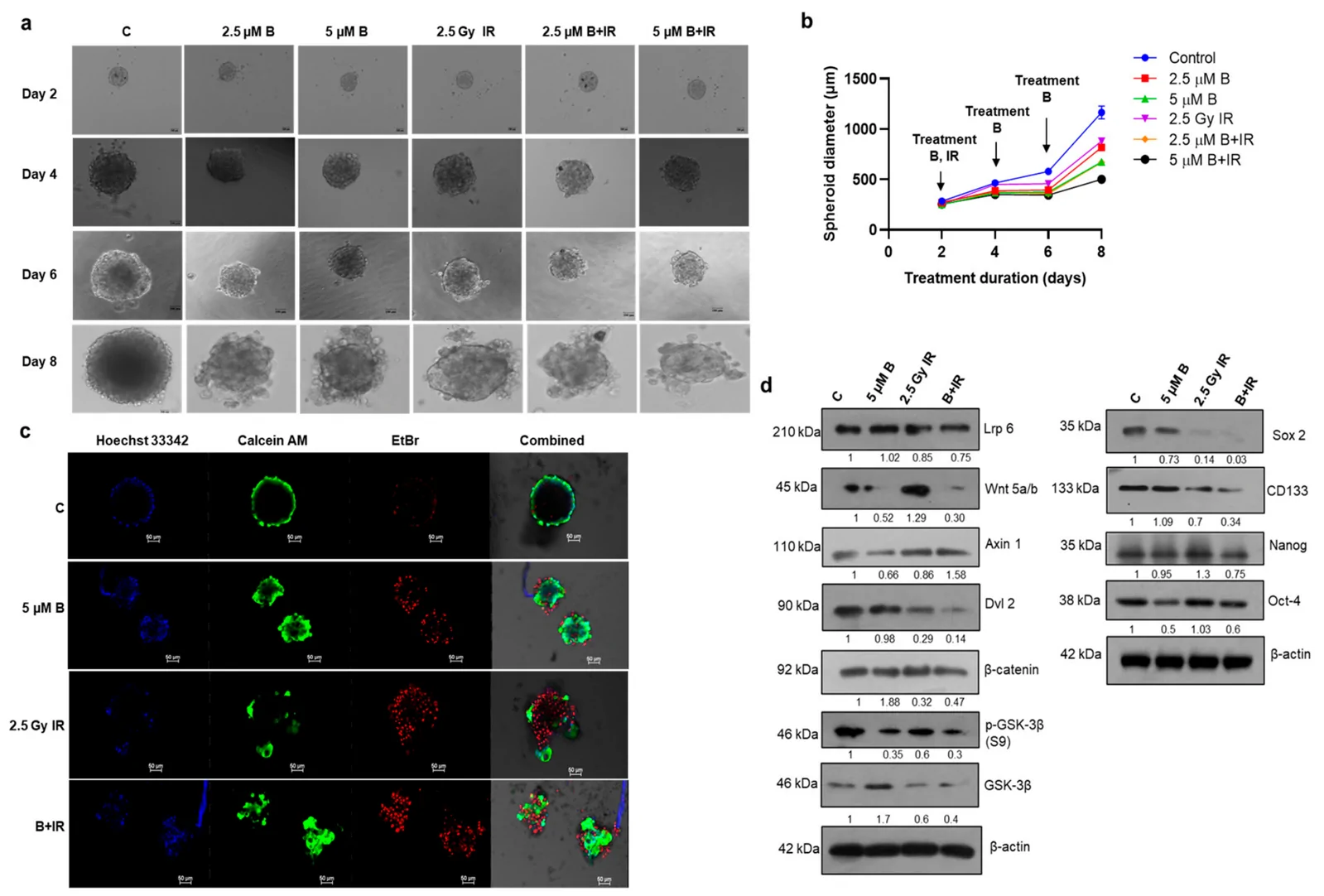
Combination of berberine and radiation decreases stemness-related features in human HNSCC and mouse OSCC cells. (**a**) UM-SCC-22B cells (300 cells/well) were seeded on an agarose-coated 96-well plate to facilitate the formation of multicellular spheroids. After 48 h, these spheroids were subjected to treatment with 2.5 and 5 µM berberine, either alone or in combination with 2.5 Gy radiation, and were observed over a period of eight days. The figure presents representative microscopic images of the spheroids captured at a magnification of 100×. (**b**) The growth kinetics of spheroids are indicated by the diameter of the spheroids. (**c**) Representative confocal images of spheroids, Calcein-AM (indicating live cells in green), Ethidium Bromide (indicating dead cells in red), and Hoechst-33342 (staining nuclei in blue) were employed for spheroid staining. (**d**) Western blot analysis for key proteins involved in maintaining stemness properties after treatment with berberine and/or IR.

We checked for the spheroid formation upon treatment with berberine and IR. Berberine (5 µM) and 2.5 Gy IR treatment alone led to 43% (*p* < 0.001) and 24% (*p* < 0.001) decreases in the spheroid diameter on day 8. The combination of 2.5 µM berberine and 2.5 Gy IR reduced the spheroid diameter by 43% (*p* < 0.001), which was to the extent of 5 µM berberine treatment alone. However, 5 µM berberine and 2.5 Gy IR led to a much greater decrease in the spheroid diameter by 57% (*p* < 0.001) (Figure 4a,b). In addition to the reduction in spheroid diameter, spheroid volume was also reduced. Also, berberine and IR-treated spheroids displayed signs of structural disintegration, with the cells dissociating from the main spheroid body (Figure 4c).

Anchorage-independent growth was then analyzed upon berberine and/or IR treatment, and a decrease in the colony number was observed in UM-SCC-22B cells (Figure S2b,c). To assess the nature of cells dissociating away from the spheroids, live–dead cell staining was performed. Control and treated spheroids were stained on day 9 with Calcein AM (live cells, green), EtBr (dead cells, red), and Hoechst 33342 (nuclei, blue), and visualized by confocal microscopy. In control spheroids, viable cells were primarily located at the periphery, whereas IR and/or berberine-treated spheroids displayed live cells throughout the spheroid body. EtBr staining revealed dead or dormant cells concentrated at the core of control spheroids and among the detached cells in treated groups (Figure 4c). These results confirmed that the dissociating cells after combined berberine and IR treatment were predominantly non-viable.

Since the Wnt/β-catenin pathway is mainly involved in regulating the stemness properties, we then assessed the key proteins of this pathway. We found downregulation of Wnt-5a, a non-canonical Wnt ligand that can also decrease β-catenin levels, thereby shutting down this pathway. Axin 1 acts as a negative regulator by forming the destruction complex, thereby leading to the degradation of β-catenin. The levels of Axin 1, a known negative regulator of β-catenin, were upregulated after berberine and IR treatment. Dishevelled-2, which is known to modulate GSK-3β, was downregulated along with GSK-3β, which further confirms impairment of the Wnt/β-catenin pathway (Figure 4d and Figures S12 and S13: Uncropped Western blot images).

We further observed the downregulation of pluripotency-related transcription factors, Oct4, Sox2, and Nanog, in the berberine and IR treatments. CD133, a marker for cancer stem cells, was also decreased (Figure 4d). Overall, these results indicated that berberine, along with IR, resulted in decreased stemness-related features with impairment in the Wnt/β-catenin signaling pathway in cancer cells.

### 3.5. Berberine Potentiates IR-Induced DNA Damage and Apoptosis in EGFR-Knockdown Cells

**Figure 5:**
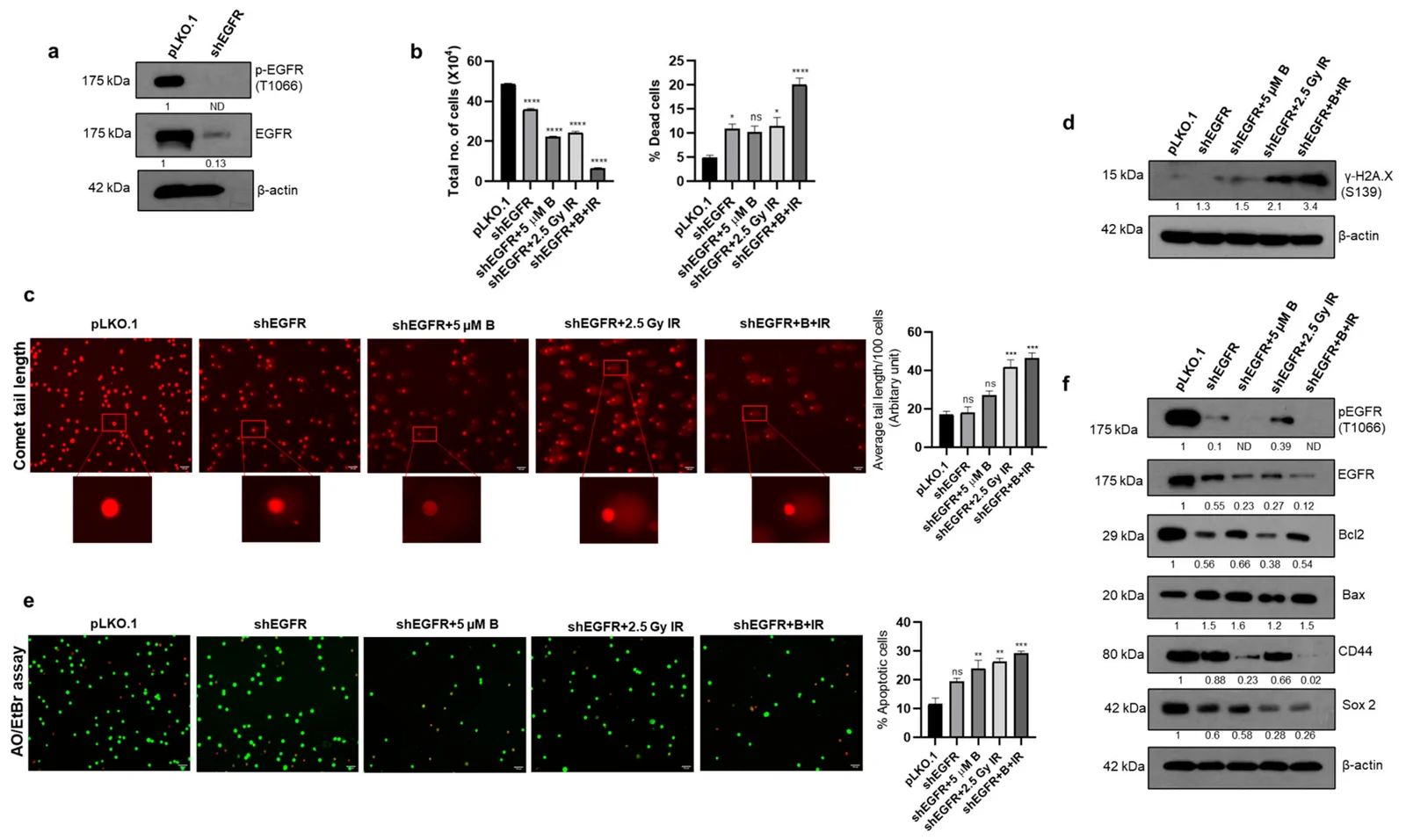
Effect of berberine and IR on EGFR-knockdown condition on cell proliferation, DNA damage, stemness and apoptosis. (**a**) Knockdown efficiency of shRNA against EGFR in UM-SCC-22B cells was evaluated at the protein level. (**b**) pLKO.1 and shEGFR cells (4 × 10^4^ cells/well) were seeded in a 12-well plate, and EGFR-knockdown cells were further treated with 5 µM berberine along with/or 2.5 Gy radiation, and a trypan blue assay was performed. (**c**) The comet assay was conducted as outlined in Section 2. Representative images illustrating different treatments were captured at a magnification of 100×, with the data presented as the percentage of comet tail length. (**d**) Western blot depicting the expression of γ-H2A.X. The loading control used was β-actin. (**e**) AO/EtBr was performed as described in Section 2, and percent apoptotic cells was plotted. (**f**) Western blots depicting the expression of p-EGFR, EGFR, Bax, Bcl-2, CD44, and Sox2. *, *p* < 0.05; **, *p* < 0.01; ***, *p* < 0.001; ****, *p* < 0.0001.

We studied the effects of berberine and IR in EGFR-knockdown UM-SCC-22B cells, generated via shRNA-mediated EGFR-knockdown. The confirmation of knockdown efficiency was achieved by observing the reduction in the levels of phospho-EGFR and total EGFR protein. The EGFR-knockdown UM-SCC-22B cells showed an 87% decrease in the protein level of total EGFR expression, while no band was detected for the phosphorylated EGFR as compared to the vector control (Figure 5a and Figure S14: Uncropped Western blot images). The EGFR-knockdown cells showed a decrease in the total number of cells by 26% (*p* < 0.001) and an increase in the dead cells from 5% in pLKO.1 vector control to 20% (*p* < 0.001) in knockdown cells. The total number of cells was reduced by 54% (*p* < 0.001) with the administration of 5 µM berberine alone, which was further decreased by 86% (*p* < 0.001) when combined with 2.5 Gy IR (Figure 5b).

EGFR-knockdown UM-SCC-22B cells exhibited DNA damage, which was further augmented upon berberine (5 µM) treatment. Interestingly, the combination of berberine with 2.5 Gy IR resulted in an escalation of DNA damage, exceeding the individual impacts of each treatment. This was evidenced by an increase in comet tail length from 17 a.u. in the pLKO.1 vector control to 18 a.u. (*p* = ns) in EGFR-knockdown cells, and reaching 27 a.u. in EGFR-deficient cells upon treatment with berberine. The combination of berberine and IR further amplified this effect, resulting in a comet tail length of 46 a.u. (*p* < 0.001) (Figure 5c) alongside an increased expression of γ-H2A.X (Ser139) (Figure 5d). This highlights the potential role of EGFR signaling in the DNA damage response and suggests that blocking EGFR signaling makes cells more sensitive to genotoxic stress. Moreover, berberine and IR also target mechanisms other than EGFR signaling in these cancer cells.

The apoptosis analysis provided further evidence for the increased effectiveness of the combined treatment. The percentage of apoptotic cells increased from 12% in the pLKO.1 vector control to 19% in EGFR-deficient HNSCC cells. Following treatment with berberine, the apoptotic cells increased to 24% (*p* < 0.01), which subsequently increased to 29% (*p* < 0.001) when combined with both berberine and IR (Figure 5e). The expression levels of Bax and Bcl-2 were comparable in both EGFR-knockdown cells and knockdown cells treated with berberine and IR, suggesting that the pro-apoptotic effects of berberine may, at least in part, be mediated through inhibition of EGFR signaling (Figure 5f and Figure S15: Uncropped Western blot images).

The expression of CD44, a marker associated with cancer stem-cell-like properties, decreased following EGFR knockdown and was further diminished by treatment with berberine and was even more reduced when combined with IR. This indicates the involvement of alternative pathways, aside from EGFR, in regulating berberine and IR-mediated DNA damage response and stemness characteristics in cancer cells (Figure 5f).

### 3.6. Berberine and IR Inhibited Tumor Growth in the Syngraft Mouse Model

**Figure 6:**
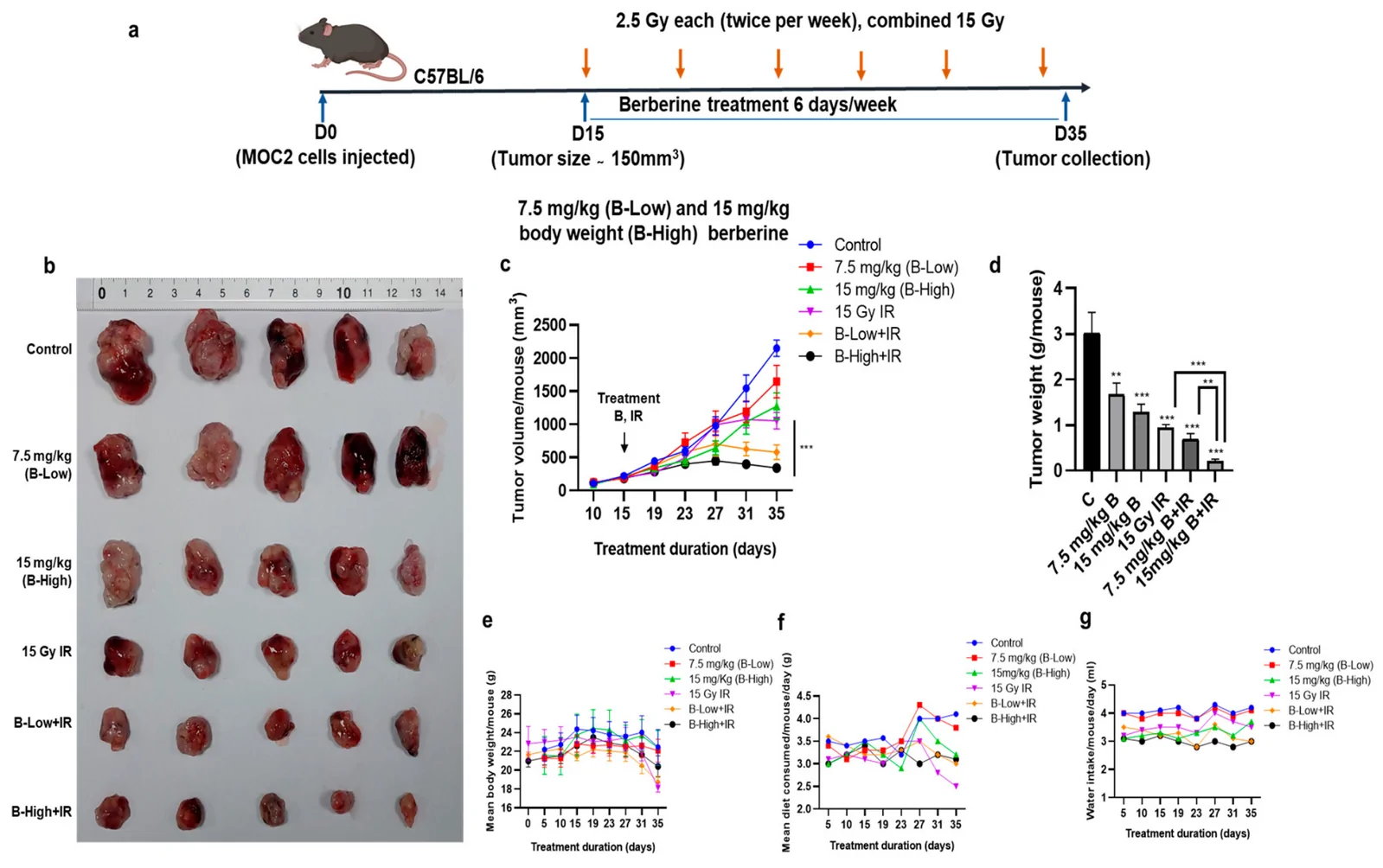
Effect of berberine and radiation on tumor growth in mice. (**a**) Timeline illustrating the procedure followed in the tumor study. MOC2 cells (1 × 10^5^) were injected subcutaneously in C57BL/6 mice, in combination with Matrigel (1:1 ratio) and observed until tumor volume reached 150–200 mm^3^. Mice were then randomized and treated with 7.5 and 15 mg/kg berberine, given 6 days per week along with/ or 2.5 Gy biweekly dose of radiation until a combined dose of 15 Gy is achieved. The control and IR group were treated with 0.9% saline. (**b**) Representative images of tumors at the end of the treatment. (**c**) Quantitative data representing tumor volume/mouse during the course of treatment. (**d**) Representative graph showing mean tumor weight/mouse in mg at the end of the study. (**e**–**g**) Representative graphs of mean body weight/mouse, mean diet and water consumption/mouse throughout the course of treatment. **, *p* < 0.01; ***, *p* < 0.001.

**Figure 7:**
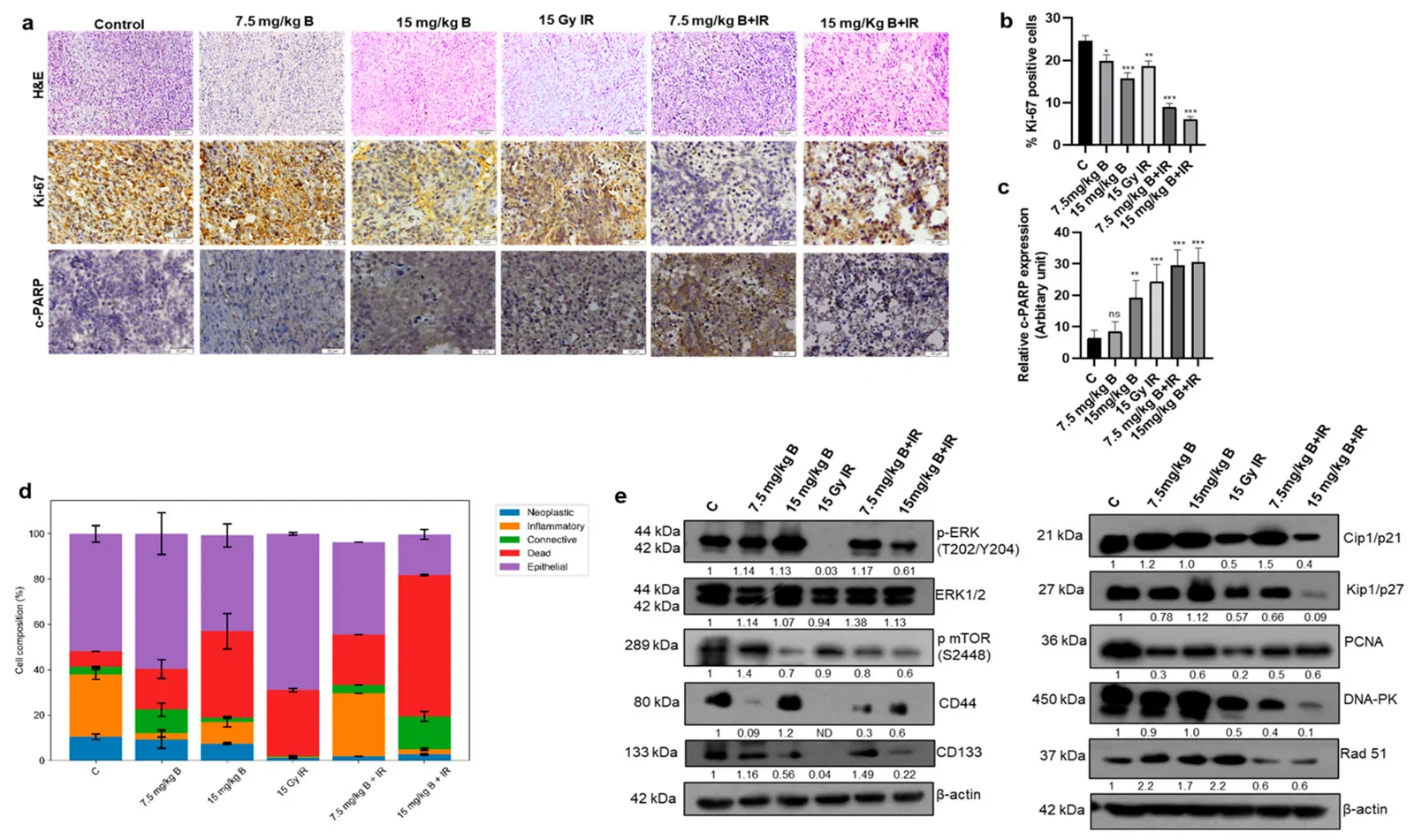
The effect of berberine and radiation treatment on cell proliferation and apoptosis in MOC2 syngrafts. Following the treatment, the tumors were surgically removed, sectioned, and subjected to H&E and IHC staining. (**a**) Representative images of H&E staining to assess histological morphology and IHC staining for Ki-67 and cleaved PARP-positive cells in berberine and/or radiation-treated tumors at 100× magnification. (**b**,**c**) Graph depicting percentage of Ki67 and cleaved PARP-positive cells. (**d**) Quantitative distribution of different cell types identified by HoVer-Net-based classification of H&E-stained images as mentioned in Materials and Methods. The relative percentage of each cell type is shown as stacked bar graphs across treatment groups. (**e**) Western blot analysis of tumor tissues for p-ERK1/2, ERK1/2, p-mTOR, p21^Cip1^, p27^Kip1^, PCNA, CD44, CD133, DNA-PK and Rad 51 expression. β-actin was used as a loading control. *, *p* < 0.05; **, *p* < 0.01; ****, *p* < 0.0001; ns, non-significant.

To determine the *in vivo* effects of berberine in combination with IR, we utilized a syngeneic MOC2 mouse model. Mice with tumors measuring a volume of approximately 150 mm^3^ were randomized to receive treatment with berberine at doses of 7.5 mg/kg and 15 mg/kg, either alone or in combination with IR over the treatment duration. Berberine was administered via oral gavage six days per week, while IR was delivered twice weekly at a dose of 2.5 Gy per dose, resulting in a cumulative dose of 15 Gy (Figure 6a). Consistent with the *in vitro* data, the tumor volume was reduced by 23% (*p* = ns) and 41% (*p* < 0.001) as compared to control tumors upon treatment with either a low or high dose of berberine, respectively, and along with a 51% (*p* < 0.001) reduction with IR-alone treatment. However, in combination with IR, the 7.5 mg/kg and 15 mg/kg berberine led to 73% (*p* < 0.001) and 84% (*p* < 0.001) decreases in tumor volumes as compared to the control on day 35 (Figure 6b,c). On the 27th day after syngraft implantation, the combination treatments of both doses of berberine with IR halted the tumor growth and demonstrated the regression of tumors during the study period. Consequently, the tumor weight at the end of the treatment decreased to 0.205 g/tumor in the berberine high-dose combination with IR group, a 93% (*p* < 0.001) decrease, compared to 3.024 g/tumor in the untreated control group (Figure 6d). Also, the H&E staining demonstrates that the reduction in tumor size is accompanied by a marked decrease in tumor cell density and disruption of tumor architecture compared with the control group (Figure 7a). The evaluation of H&E-stained tumor sections revealed treatment-induced changes that were consistent with the deep learning-based cell type analysis (Figure 7d, Figures S16 and S17: Uncropped Western blot images). Control tumors showed an abundance of neoplastic cells as per the PanNuke classification system. Berberine treatment induced moderate histological changes with a slight reduction in neoplastic cells, whereas IR led to a reduction in neoplastic cells and a relative increase in connective tissue and dead-cell populations. These effects were more enhanced in the 15 mg/kg berberine with IR treatment group, which exhibited architectural disruption, loss of viable tumor cells, and stromal expansion. This was consistent with the highest abundance of dead cells and the lowest proportion of neoplastic cells identified by HoVer-Net. Together, these findings indicate that berberine enhances the effect of IR by promoting tumor cell death and tissue remodeling while reducing the viable tumor cell population. One of the side effects of IR is a reduction in body weight, which was 18.14 g in the IR-treated group as compared to 22.46 g in the control group at the end of the treatment duration. Nevertheless, this reduction was mitigated when berberine was administered alongside IR, resulting in a body weight increase to 20.4 g (Figure 6e), indicating a possible protective effect of berberine against the systemic effects induced by IR. Additionally, dietary intake, similar to body weight, was better in the berberine high-dose and IR group and water consumption remained largely unchanged (Figure 6f,g).

### 3.7. Berberine and IR Decrease Proliferation and Induce Apoptosis in MOC2 Tumors

Tumors were fixed and sectioned for hematoxylin and eosin (H&E) staining, and immunohistochemistry (IHC) was performed for detecting the protein expression levels of Ki-67 and cleaved PARP. The Ki-67-positive cells, which is a proliferation marker, decreased to 8.9% (*p* < 0.001) and 6% (*p* < 0.001) in 7.5 mg/kg and 15 mg/kg berberine along with IR, respectively, as compared to 24.5% in the control group (Figure 7a,b). The percentage of cells positive for cleaved PARP, a known marker of apoptosis, rose to 29% (*p* < 0.001) and 31% (*p* < 0.001) in the groups treated with 7.5 mg/kg and 15 mg/kg of berberine, respectively, along with IR treatment, in contrast to only 6% observed in the control group (Figure 7a,c). These results further substantiate the hypothesis that berberine, when used along with IR, inhibits tumor cell proliferation and promotes apoptosis, thereby contributing to the observed decrease in the kinetics of the tumor growth and subsequently leading to the regression of tumors.

We then assessed the expression of key pro-survival molecules, namely phosphorylated mTOR and phospho-ERK1/2, in mouse tumors through immunoblotting. The expression levels of these proteins were reduced in response to 15 mg/kg of berberine in combination with IR exposure. The downregulation of PCNA, a marker associated with cell proliferation, corresponds with the reduction of Ki-67-positive cells observed in IHC analysis, suggesting a decrease in cellular proliferation following treatment with berberine and IR. Furthermore, both Rad 51 and DNA-PK, which are crucial components of the HR and NHEJ pathways, exhibited downregulation following treatment with 15 mg/kg of berberine and IR, suggesting a disruption in the DSB repair pathway proteins (Figure 7e, Figures S16 and S17: Uncropped Western blot images and Figure S3). The reduction of CD44 and CD133 levels following treatment with 15 mg/kg of berberine and IR aligns with the observed decrease in spheroid growth and stemness properties (Figure 4a). Overall, these findings suggest that berberine, when combined with IR, disrupts oncogenic signaling, impairs DNA repair proteins, and decreases stemness-related markers in cancer cells.

## 4. Discussion

The study presents several key findings, including berberine inhibiting cell proliferation in both human HNSCC and mouse OSCC cells, inducing a sub-G1 phase arrest, promoting apoptosis, increasing the ratio of Bax to Bcl-2 as well as the levels of cleaved caspase 3 and cleaved PARP, and also inhibiting mitogenic and pro-survival signaling. Further, berberine potentiated IR-induced DNA damage and altered the expression of key proteins associated with DNA repair, reduced stemness-associated features, and changed the morphology of spheroids, leading to distortion in their shape, with the cells detaching from the main body of spheroids, with downregulation of the Wnt/β-catenin signaling and pluripotency factors. Berberine enhanced IR-induced DNA damage and reduced stemness in EGFR-knockdown cells. *In vivo*, berberine attenuated the growth of syngeneic mouse tumors and, in combination with IR, demonstrated tumor regression after day 27. Berberine also decreased expression of stemness-related markers, increased apoptosis, and reduced proliferation and pro-survival signaling in tumors, thereby corroborating the effects observed in *in vitro* cell culture. Overall, our findings indicate that berberine functions to enhance the radiation response in both human HNSCC (UM-SCC-22B) and mouse OSCC (MOC2) cell lines and animal tumor model, and significantly amplified radiation-induced cell death through multiple mechanisms.

Studies have demonstrated that berberine possesses anticancer properties across multiple tumor types, such as colon, breast and prostate cancers. Its potential in enhancing the sensitivity of HNSCC to radiation therapy remains inadequately explored. In the present study, berberine enhanced radiation-induced inhibition of cell proliferation in both HNSCC and OSCC cells. This observation aligned with the reduced expression of PCNA in both cell lines. These findings are supported by earlier studies where berberine radiosensitized ovarian cancer cells [12] and prostate cancer cells [29] to radiation.

Radiation-induced survival signaling is a key contributor to tumor cell survival following radiation treatment, and inhibiting these pathways may offer an effective strategy to overcome radioresistance. We found the downregulation of EGFR signaling along with decreased expression of p-ERK1/2, p-STAT3, and p-mTOR. Berberine has previously been shown to reduce EGFR-ERK signaling in glioblastoma cells [30], as well as in gastric cancer [31]. Furthermore, apoptosis is a key mechanism for enhancing the therapeutic response to the treatment. Berberine is shown to induce apoptosis in cancer cells through modulation of mitochondrial and MAPK signaling pathways by promoting reactive oxygen species (ROS) generation, leading to oxidative stress and activation of the apoptotic cascade [32]. Also, the increase in sub-G1 cells is based on the idea that fragmented low-molecular-weight DNA is liberated from cells following DNA endonucleolytic cleavage during extended fixation. This will produce a population of cells that binds the quantitative DNA stain, PI, to a lesser degree than is normal for G1 cells. This cell population will therefore be located to the left of the G1 peak. As expected, in our study, berberine caused an increase in sub-G1 cells as well as apoptotic cells, with an increased ratio of pro-apoptotic Bax to anti-apoptotic Bcl2 levels, along with elevated levels of cleaved caspase 3 and cleaved PARP, and more so in combination with IR suggests the role of berberine as a potential adjuvant for the radiation therapy of HNC. Both the human HNSCC and mouse OSCC cell lines demonstrated a consistent response to berberine and/or radiation treatment. However, differences in the magnitude of the response were observed, highlighting the biological heterogeneity between the two models.

While looking at the molecular evidence, we observed an elevated expression of phosphorylated p53, a key regulator of DNA-damage-induced apoptosis, following IR treatment, which was further enhanced when combined with berberine. This finding corroborated the observed reduction in MDM2 levels, a known inhibitor of p53. Nevertheless, it is important to note that UM-SCC-22B cells harbor mutations in p53, rendering it inactive [33]. However, further studies are needed to explore whether berberine reactivates p53 for its tumor suppressor function as reported earlier with PRIMA-1 or CP-31398 [34]. A decreased expression of cell cycle inhibitors p21/cip1 and p27/kip1 was observed, owing to the decreased stability and metabolic changes, which may trigger apoptotic cell death [35].

Radiation therapy relies on damaging the DNA of cancer cells beyond the threshold of the repair capacity of these cells. However, it requires higher doses of radiation exposure, which also causes death of normal cells and thus limits the success of the therapy. Our data suggested that berberine-caused increase in IR-induced DNA damage could enhance the therapeutic response. This was also supported by the decreased expression of key proteins in NHEJ and HR DNA repair pathways. IR is known for its ability to cause DSBs, which are primarily repaired through the NHEJ mechanism [36]. The reduction in the expression level of DNA-PK, a crucial protein involved in the NHEJ pathway, alongside a decrease in Ku70, further supported this assumption at the molecular level. Further, a decrease in the expression of Rad51, a key protein involved in the HR repair pathway, suggested a potential disruption in DNA repair pathway-related proteins following berberine and IR treatment. Further, elevated levels of ATM/CHK2 also suggested an increase in DSB resulting from the combined treatment of berberine and IR [37]. Overall, these findings suggested the downregulation of DNA repair proteins, leading to enhanced DNA damage in cancer cells by the combination treatment.

Cancer stem cells are characterized by self-renewal and pluripotency and play an important role in metastasis, tumor relapse, and therapy resistance. Cancer stem cells also exhibit resistance to radiation, making their targeting a viable strategy to enhance radiation sensitivity [38]. The multicellular tumor spheroids (MCSs) mimic the complex cell–cell adhesion and cell–matrix interactions in solid tumors, leading to the development of metabolite gradients for nutrition and growth factor signals as observed in vivo. Due to the metabolite gradient and a complex microenvironment, MCSs exhibit proliferative, quiescent, and necrotic zones, similar to the internal milieu of human tumors. Furthermore, due to their multicellular composition, MCSs inherently acquire multidrug resistance to several chemotherapeutic agents, rendering them an ideal model system for this investigation [39,40]. The spheroid growth kinetics findings suggested that berberine impairs the growth of spheroids and more so along with IR treatment, suggesting a potential targeting of cancer stem-like cells and enhancing sensitivity to IR. The combination of berberine and IR also distorted the shape, showing disintegration of spheroids. The cells began to separate from the primary structure of spheroids, and the properties of these detached cells showed a deceased state, ruling out the possibility of cellular metastasis after shedding these cells upon the combination treatment [41,42]. This is supported by an earlier report, wherein berberine along with radiation decreased the viability of MCF-7 spheroids. Berberine alone has also been shown to suppress spheroid formation in lung cancer and in Pancreatic Ductal Adenocarcinoma when combined with Gemcitabine [43,44].

Wnt-5a has been positively correlated with and implicated in inflammatory diseases, including tumor promotion and progression. A downregulation of Wnt-5a by berberine, a non-canonical Wnt ligand, supported the anti-inflammatory and anticancer activities of berberine. Further, Axin (Axis Inhibitor Protein) 1, a negative regulator, forms a destruction complex that facilitates the degradation of β-catenin. An upregulation of Axin 1 level was observed following treatment with berberine and radiation, suggesting the downregulation of the β-catenin pathway. Additionally, Dishevelled-2, which is known to modulate GSK-3β, was downregulated alongside GSK-3β, further supporting the notion of Wnt/β-catenin pathway impairment. A noted decrease in pluripotency markers, such as Oct4, Sox2, and Nanog, under berberine and radiation conditions, as well as a reduction in CD133, a marker indicative of cancer stem cells, provides evidence for a decrease in stemness-related properties in the combination treatment. Collectively, these findings suggest that the combination of berberine and IR leads to a reduction in stemness-associated properties, accompanied by a disruption of the Wnt/β-catenin signaling pathway.

EGFR is known for its role in the regulation of DNA damage repair, cellular survival, and the maintenance of stem cell properties [45,46], as also observed in the present study. Berberine treatment in the EGFR knockdown condition, as anticipated, increased DNA damage and inhibited stemness, which was further amplified when combined with ionizing radiation (IR). This indicates the involvement of alternative pathways, aside from EGFR, in the modulation of these characteristics by berberine and radiation. Conversely, the levels of Bax and Bcl2 expression were similar in both the EGFR knockdown and the berberine treatment following EGFR knockdown, as well as in conjunction with IR, indicating the involvement of EGFR in the regulation of berberine-induced mitochondrial apoptosis.

The syngraft studies in mice demonstrated that the administration of berberine at dosages of 7.5 and 15 mg/kg, in combination with radiation therapy, was effective in reducing both tumor volume and tumor weight. The reduction in tumor size was accompanied by histopathological alterations that were corroborated by computational cell type analysis, indicating increased tumor cell death and reduced tumor cellularity. These findings were also supported by deep learning-based cellular profiling, which indicated that berberine potentiated the effects of IR by reshaping the tumor cellular landscape. The reduction in neoplastic cells with the increase in dead cells after berberine and IR treatment suggests enhanced treatment-induced tumor cell death. The concomitant increase in connective tissue cells likely reflects tissue remodeling following tumor regression. These observations are consistent with our immunohistochemical findings, resulting in reduced Ki-67 and increased cleaved PARP expression, further supporting that berberine augments the therapeutic efficacy of IR by suppressing tumor cell survival and promoting cell death.

The enhanced in vivo efficacy of berberine is likely due to the contribution of various mechanisms that are absent in conventional cell culture. In addition to radiosensitizing tumor cells through increased oxidative stress, DNA damage, and apoptosis, berberine has been shown to modulate the tumor microenvironment by promoting antitumor immunity, suppressing immunosuppressive macrophage polarization, and inhibiting angiogenesis through downregulation of VEGF and HIF-1α signaling [47,48]. These mechanisms synergize with radiation-induced cell death, leading to more effective elimination of tumor cells and sustained suppression of tumor growth in vivo. Moreover, the in vivo endpoints, including tumor volume and tumor weight, represent the cumulative effects of repeated treatment on tumor cell death, proliferation, angiogenesis, and host immune responses over time. In contrast, in vitro cell death assays evaluate only the direct cytotoxic effects of treatment on isolated cells, without accounting for the complex interactions within the tumor microenvironment. Consequently, a more pronounced antitumor effect is expected in vivo than in vitro.

Previous studies indicate that two weeks of oral gavage administration of berberine at a dosage of 20.8 g/kg in ICR mice did not show any fatalities in mice and was non-toxic [49]. The berberine dosages utilized in this study were also non-toxic, which also resulted in a reduction of radiation-induced toxicity, as indicated by a lesser decrease in body weight observed in the group receiving the combination therapy as compared to IR alone, which is one of the significant side effects of radiotherapy, suggesting a possible protective effect on normal tissues and decreasing the radiation-related toxicity. The elevated levels of the cleaved caspase-PARP, coupled with the reduced expression of Ki67 and PCNA, confirmed decreased proliferation and increased apoptosis in tumors. The molecular analysis of tumors for pro-survival molecules, ERK1/2, and mTOR, stemness markers, CD44 and CD133, and DNA-damage repair, DNA-PK, and RAD51 markers corroborated the similar mechanisms observed for berberine and IR from in vitro to in vivo conditions at the molecular level.

This study, however, has some limitations that should be considered. Although our findings demonstrate that berberine enhances the response of HNSCC models to radiation, radiobiological analyses, such as sensitizer enhancement ratio (SER) calculations, can further support the application of the findings. In addition, the study was limited to one human and one mouse OSCC cell line, and thus, validation in a broader panel of HNSCC models would further strengthen the findings. While the EGFR knockdown experiments suggest that the effects of berberine are not solely dependent on EGFR signaling, the involvement of other receptor tyrosine kinases and downstream signaling pathways remains to be investigated. Despite these limitations, our findings provide evidence that berberine enhances the response of HNSCC models to radiation and is associated with increased DNA damage, altered DNA damage response signaling, apoptosis, and reduced stemness-associated features, supporting its further evaluation as a potential adjuvant to radiotherapy.

**Figure 8:**
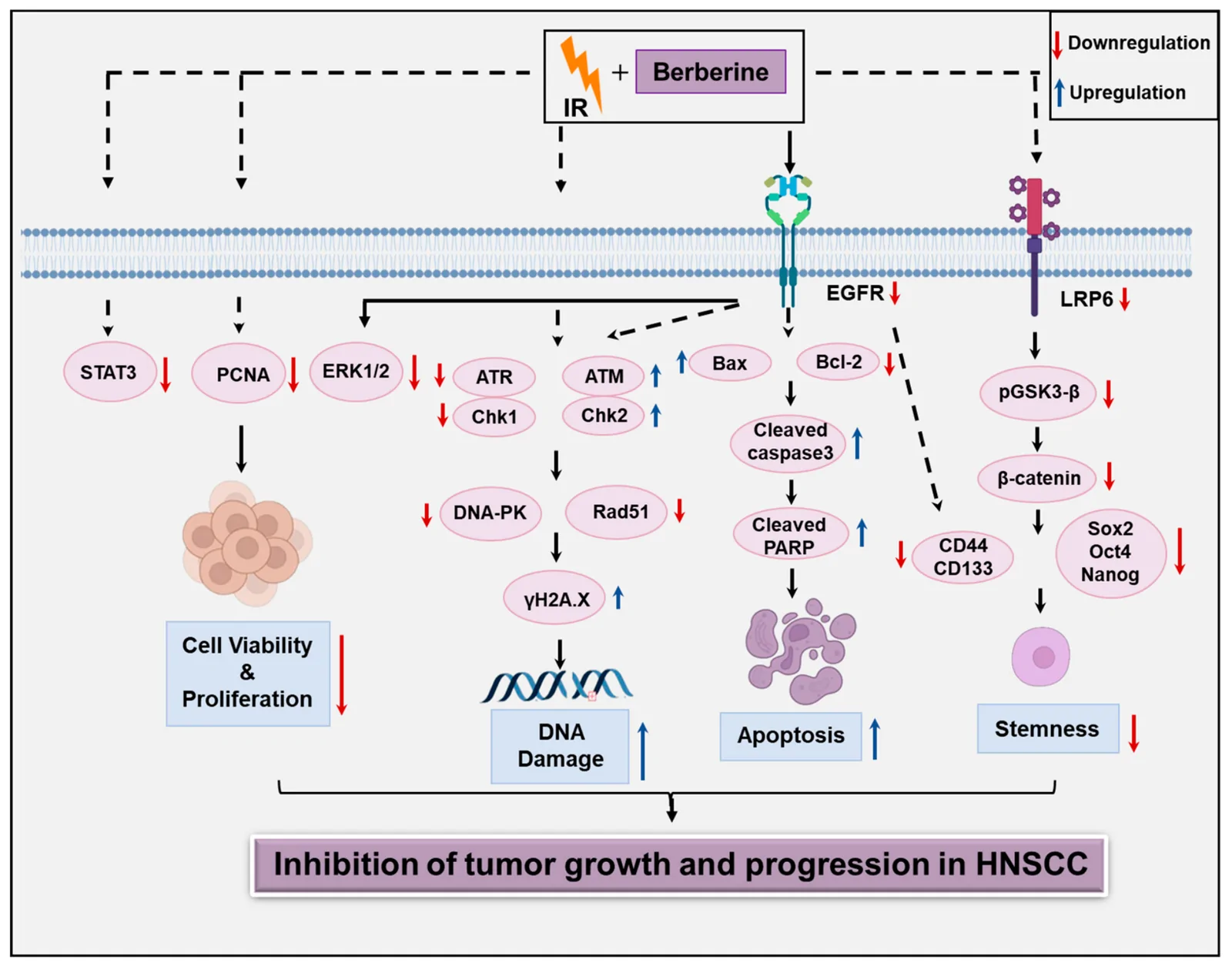
The suggested framework illustrates the effect of berberine in combination with radiation on various cellular mechanisms in human HNSCC cells. Berberine in combination with IR led to a downregulation of pro-survival signaling pathways (EGFR, Erk1/2, and STAT3) and induced apoptosis via increased cleavage of caspase 3 and PARP. The expression of key proteins associated with DNA repair (DNA-PK and Rad 51) is reduced. This combination also inhibited Wnt β-catenin signaling and reduced the expression of genes associated with stemness (CD44, CD133, Sox2, Oct4 and Nanog).

In summary, this study suggests that berberine enhances the response of head and neck cancer to radiation through modulation of pro-survival pathways, enhancement of radiation-induced cell death, DNA damage with a concomitant decrease in DNA repair, and decreased stemness properties (Figure 8). This combined treatment strategy targets and interferes with several key cancer hallmarks, such as uncontrolled proliferation and survival signaling, genomic stability maintained despite DNA damage, impaired DNA repair mechanisms, and the persistence of cancer stem cells. These findings advocate for additional investigation into the use of berberine in combination with radiation therapy approaches for head and neck squamous cell carcinoma.

## 5. Conclusions

These findings support that berberine enhances the response of HNSCC models to radiation, acting via suppression of proliferative and survival pathways, thereby limiting the ability of cancer cells to recover and proliferate following radiation exposure. Further, alteration of DNA repair pathways leads to the accumulation of unrepaired DNA damage, which ultimately enhances radiation-induced cell death. Importantly, berberine also targets stemness-associated features, a critical feature contributing to tumor resistance, recurrence, and metastasis. By attenuating stemness-related signaling, berberine diminished the self-renewal capacity and radioresistance typically observed in cancer stem-like cells. Our study provided compelling preclinical evidence that berberine enhances the therapeutic efficacy of IR in human HNSCC and mouse OSCC models. This combinatorial approach effectively disrupts multiple hallmarks of cancer, including sustained proliferation and survival signaling, genomic instability fueled by DNA damage and impaired DNA repair, and cancer stemness. These observations support the potential application of berberine in combination with radiation for HNSCC. Together, these findings highlight the translational significance of berberine for its potential use as a radiation adjuvant for patients with HNSCC.

## Data Availability

The data supporting the findings of this study are available from the corresponding author upon reasonable request.

## Supplementary Materials

The following supporting information can be downloaded at: https://www.mdpi.com/article/10.3390/cancers18162690/s1, Figure S1: Dose optimization of ionizing radiation and berberine in UM-SCC-22B cells, Figure S2: The combination of berberine and radiation reduces ROS formation and soft agar colony formation in HNSCC cells, Figure S3: The combination of berberine and radiation in MOC2 syngeneic tumors resulted in reduced expression of proliferation-associated markers and increased expression of apoptosis-related markers. Figures S4–S17: Uncropped Western blot images.

## Abbreviations

The following abbreviations were used in this manuscript.

HNC	Head and neck cancer;
HNSCC	Head and neck squamous cell carcinoma;
HR	Homologous recombination;
IR	Ionizing radiation;
B	Berberine;
NHEJ	Non-homologous end joining;
OSCC	Oral squamous cell carcinoma;
NPC	Nasopharyngeal carcinoma
ANOVA	Analysis of variance;
AO/EtBr	Acridine orange/ethidium bromide;
ATM	Ataxia Telangiectasia mutated;
ATR	ATM and Rad3-related;
BSA	Bovine serum albumin;
a.u.	Arbitrary unit;
Chk1	Checkpoint kinase 1;
Chk2	Checkpoint kinase 2;
DAB	Diaminobenzidine;
DNA-PK	DNA-dependent protein kinase;
DPX	Dibutylphthalate polystyrene xylene;
DSB	Double-strand break;
EGFR	Epidermal growth factor receptor;
Erk1/2	Extracellular signal-regulated kinase 1/2;
FBS	Fetal bovine serum;
HRP	Horseradish peroxidase;
H&E	Hematoxylin and eosin;
IHC	Immunohistochemistry;
NBF	Neutral buffer formalin;
PBS	Phosphate-buffered saline;
PCNA	Proliferating cell nuclear antigen;
PVDF	Polyvinylidene fluoride;
SD	Standard deviation;
SEM	Standard Error of Mean;
SDS-PAGE	Sodium dodecyl sulfate polyacrylamide gel electrophoresis;
SSB	Single-strand break;
STAT3	Signal transducer and activator of transcription;
IAEC	Institutional Animal Ethics Committee.
