# Supplementary material for: Berberine Enhances Radiosensitivity of Head and Neck Squamous Cell Carcinoma Concurrent with the Inhibition of DNA Repair, Stemness and Tumor Growth
Source: Cancers (Basel). 2026 Aug 19;18(16):2690. doi: 10.3390/cancers18162690 (PMC13510459; doi:10.3390/cancers18162690)
Supplement: Supplementary file 1 [file cancers-18-02690-s001.zip › cancers-4421356-supplementary.pdf]

Supplementary Figures

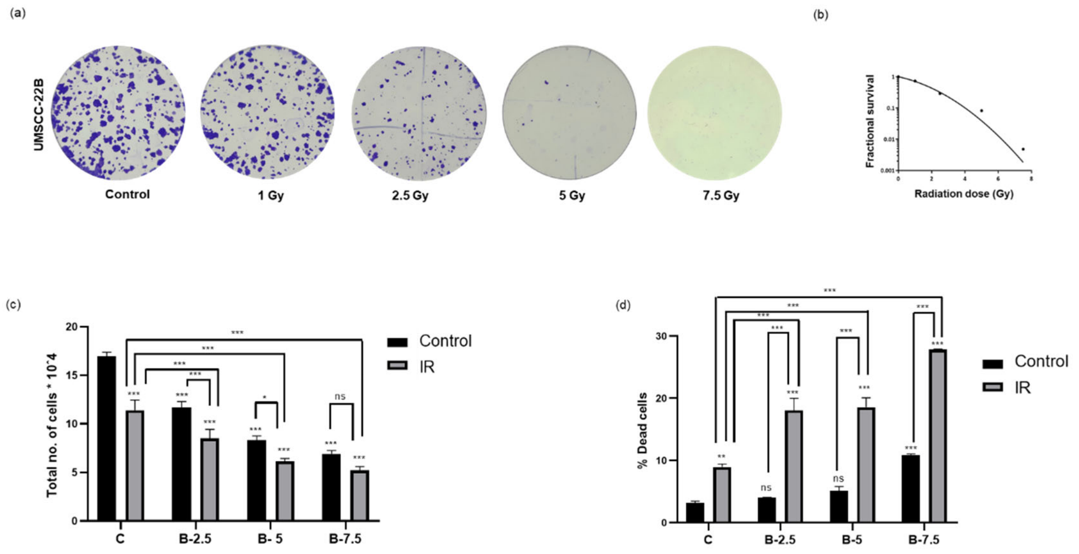

Figure S1: Dose optimization of ionizing radiation and berberine in UM-SCC-22B cells. Clonogenic assay performed on UMSCC-22B cells in response to increasing doses of ionizing radiation. Briefly, 600 UMSCC- 22B cells were seeded and subjected to a range of IR doses of 1 Gy, 2.5 Gy, 5 Gy, and 7.5 Gy and evaluated for their ability to form colonies as detailed in materials and methods. a. Representative images depicting colony formation at each radiation dose, b. The surviving fraction was quantified and plotted as a function of radiation dose to construct the survival curve. Colonies containing 50 or more cells were counted as surviving clones. UMSCC-22B cells (4×10^4) were seeded and treated with 10, 20, 2.5, 5, and 7.5 μM of berberine and/or 2.5 Gy radiation for 48 hours. The evaluation of cell viability and death was performed using the trypan blue assay, as detailed in the materials and methods section. c & d. Total number of cells, and percent dead cells after treatment with 2.5, 5, and 7.5 μM of berberine and/or 2.5 Gy radiation. *, $p<0.05$; **, $p<0.01$; ***, $p<0.001$; ns, non-significant. The p-value is computed by comparing each treatment to the control group (Two-way ANOVA). C, Control; B, Berberine; IR, Ionizing Radiation.

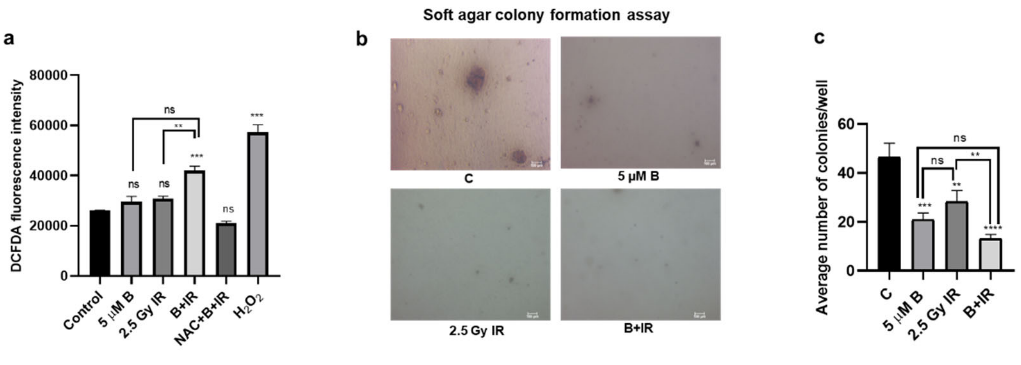

Figure S2: The combination of berberine and radiation reduces ROS formation and soft agar colony formation in HNSCC cells. a. The DCFDA assay was performed to measure ROS generation. UMSCC-22B cells were subjected to the appropriate treatment, and before this, cells were pretreated with NAC (5 mM) as detailed in the materials and methods. H_2O_2 (1 mM) served as a positive control. A representative bar graph of the 48-hour DCFDA fluorescence of UMSCC-22B. b. 2500 UMSCC-22B cells, treated with 5 μM berberine, 2.5 Gy radiation, and a combination of both, after 48 hours were collected and suspended in 2x DMEM medium and blended with agarose in a specific ratio to create a top layer over the base agarose layer, as detailed in the materials and methods section. Representative images from the soft agar assay of UMSCC-22B cells. c. A bar graph illustrating the average number of colonies per field. The results are presented as Mean ± SE from three independent samples for each treatment. **, $p<0.01$; ***, $p<0.001$; ns, non-significant. The p-value was calculated by comparing each treatment group to the control group (One-way ANOVA). C, Control; B, Berberine; IR, Ionizing Radiation; and B+IR, Combination of Berberine with Ionizing Radiation; H2O2, Hydrogen Peroxide; NAC, N-acetyl Cysteine.

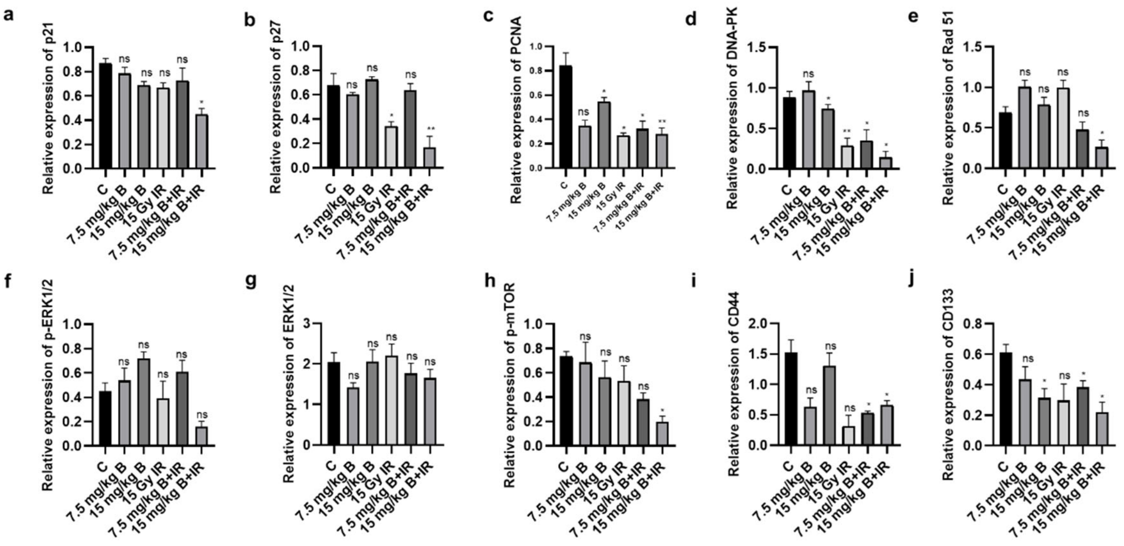

Figure S3: The combination of berberine and radiation in MOC2 syngeneic tumors resulted in reduced expression of proliferation-associated markers and increased expression of apoptosis-related markers. After completion of the treatment regimen, tumors were excised and processed for protein extraction. Western blot analysis of tumor tissues was performed to evaluate the expression levels of key signaling and stemness-related proteins. Quantitative graphs represent the relative protein expression (fold change) among the treatment groups for p-ERK1/2, ERK1/2, p-mTOR, $p21^{Cip1}$, $p27^{Kip1}$, PCNA, CD44, CD133, DNA-PK and Rad51. β-actin was used as the loading control for normalization. Protein levels were determined by densitometric analysis of Western blots performed on tumor tissues, with three independent tumors analyzed per group (n=3) *, p<0.05; **, p<0.01; ***, p<0.001; ns, non-significant.

Below are the original images of the western blots.

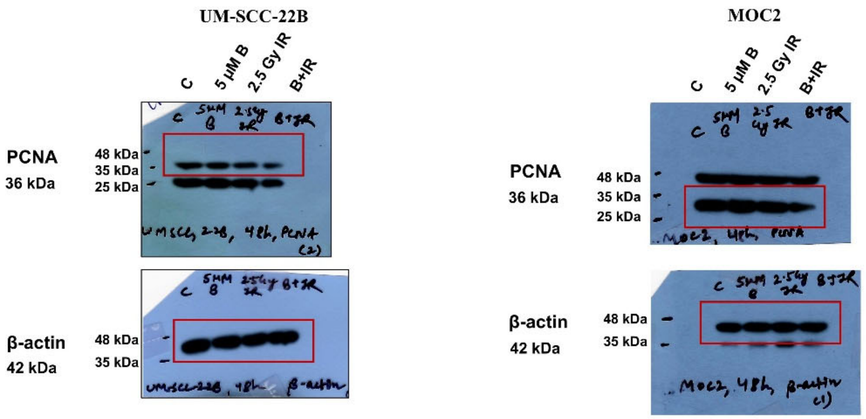

Figure S4: Original images for western blots for figure 1d (UM-SCC-22B) and f (MOC2).

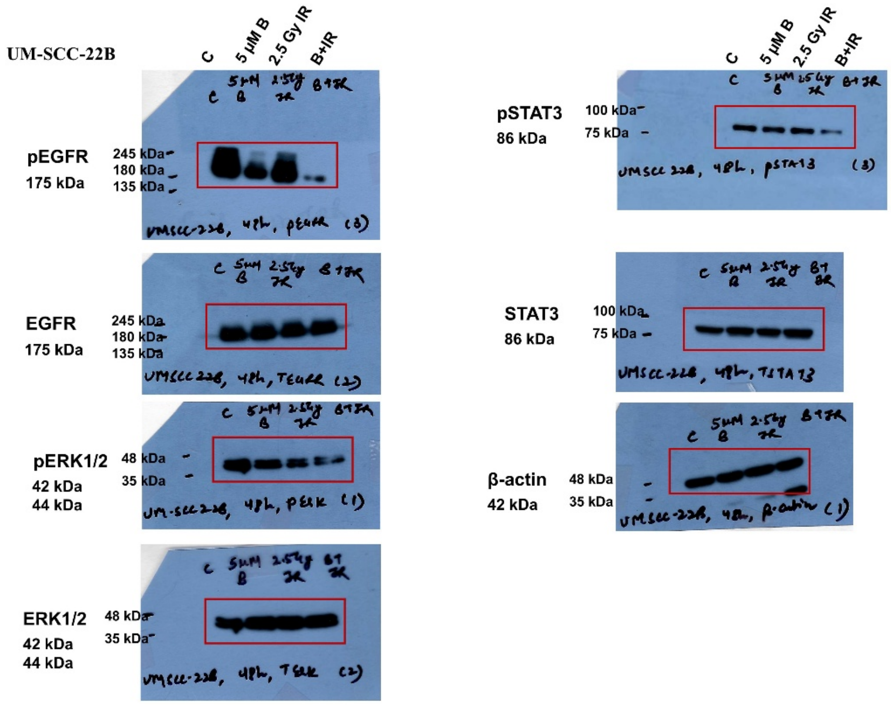

Figure S5: Original images for western blots for figure 1g (UM-SCC-22B).

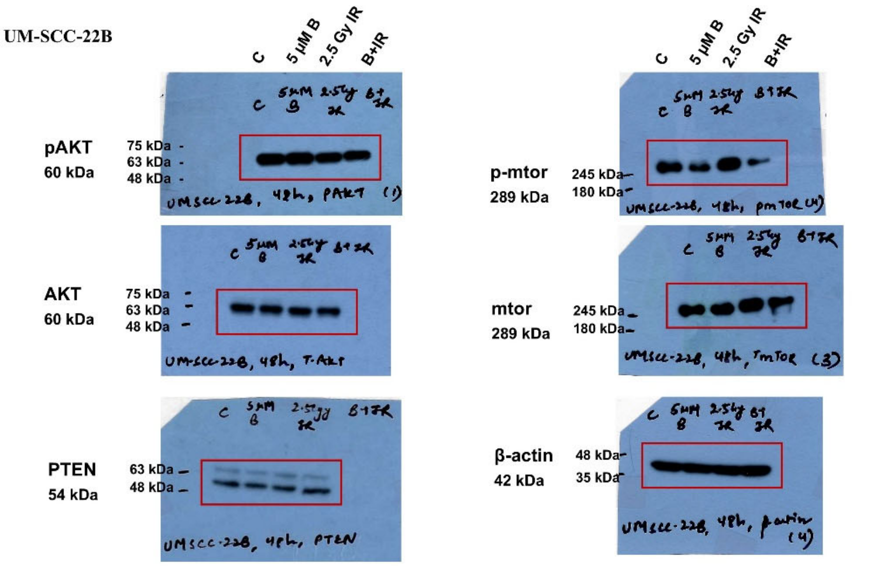

Figure S6: Original images for western blots for figure 1g (UM-SCC-22B).

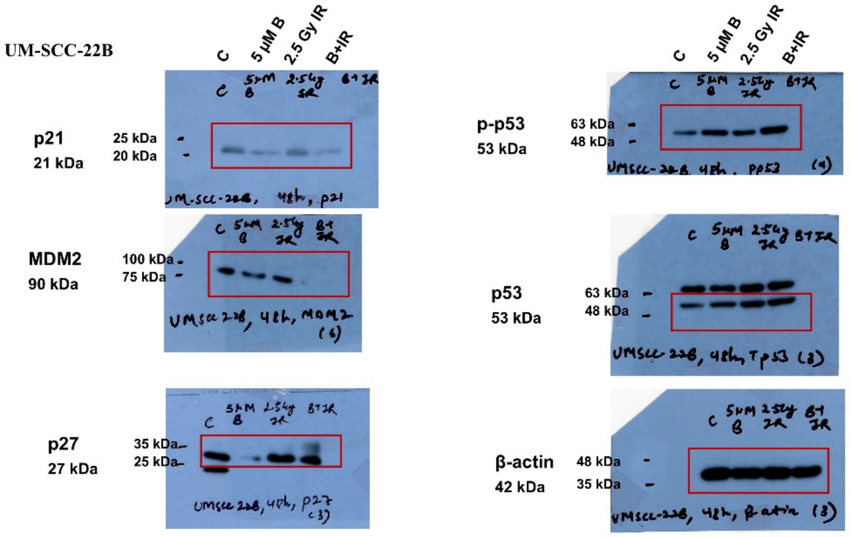

Figure S7: Original images for western blots for figure 2d (UM-SCC-22B).

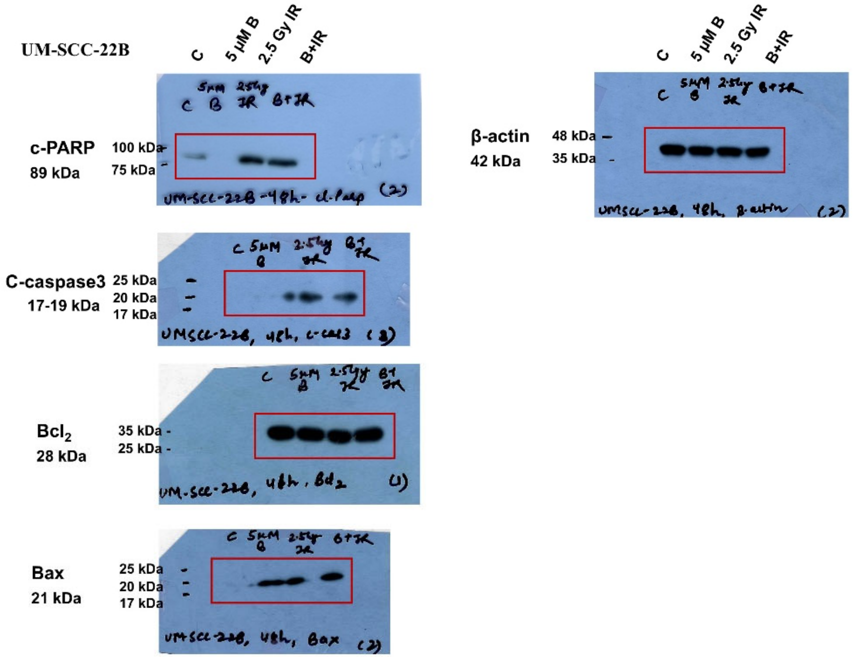

Figure S8: Original images for western blots for figure 2f (UM-SCC-22B).

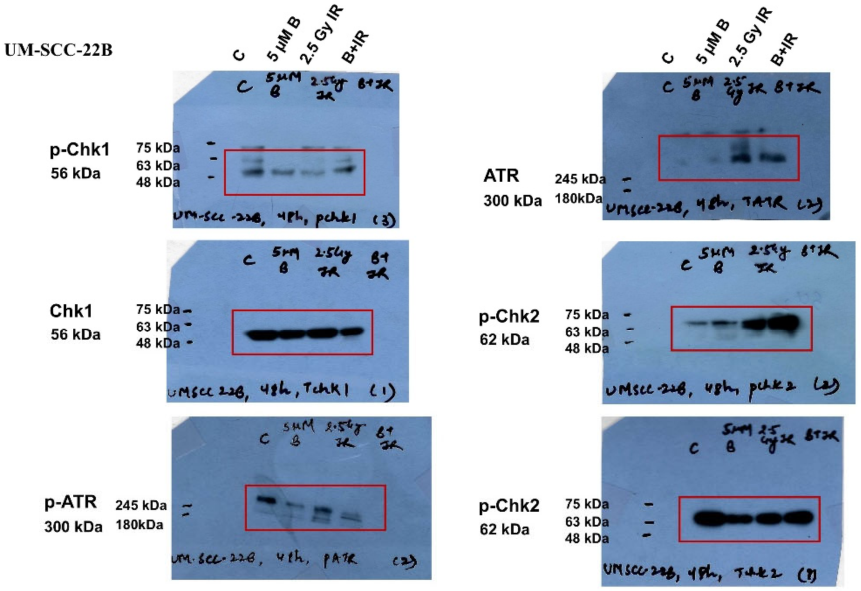

Figure S9: Original images for western blots for figure 3e (UM-SCC-22B).

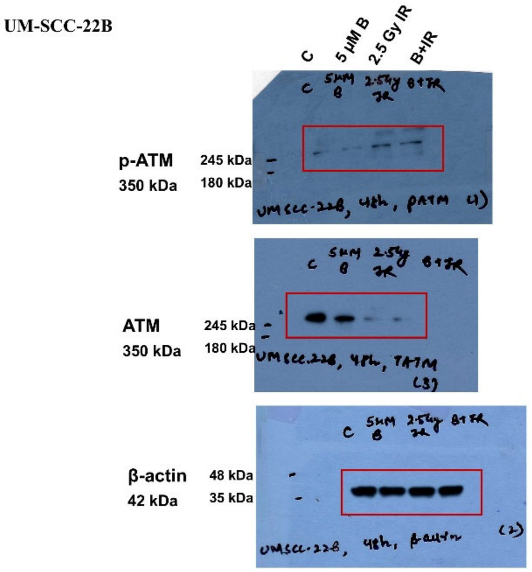

Figure S10: Original images for western blots for figure 3e (UM-SCC-22B).

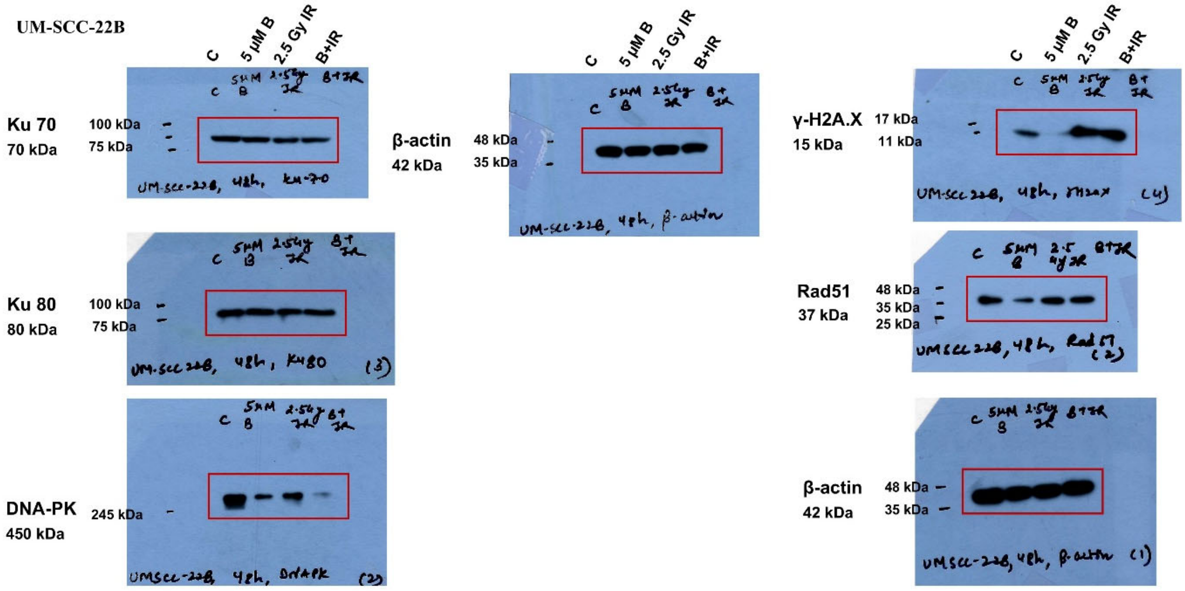

Figure S11: Original images for western blots for figure 3e (UM-SCC-22B).

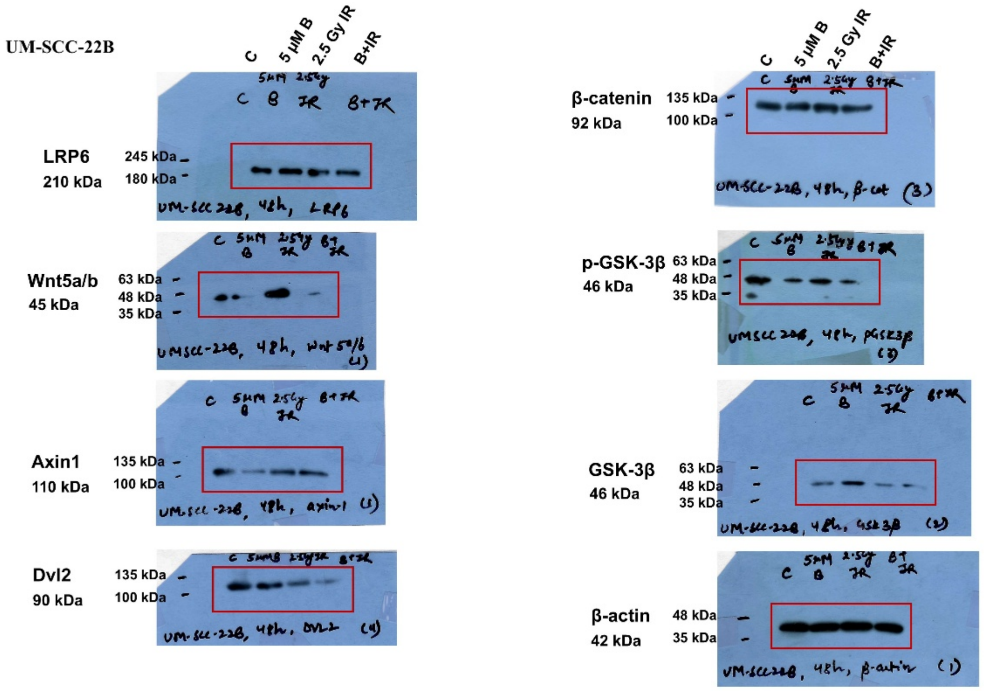

Figure S12: Original images for western blots for figure 4d (UM-SCC-22B).

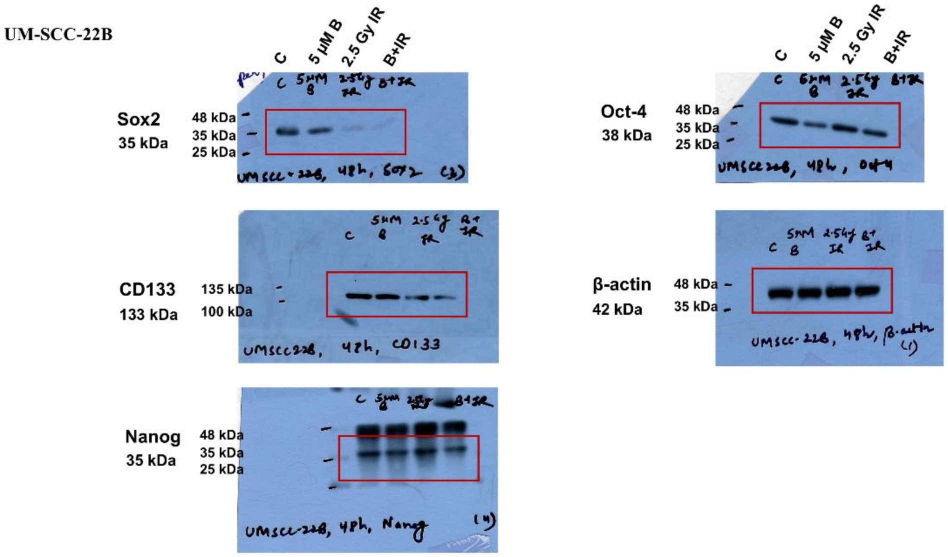

Figure S13: Original images for western blots for figure 4d (UM-SCC-22B).

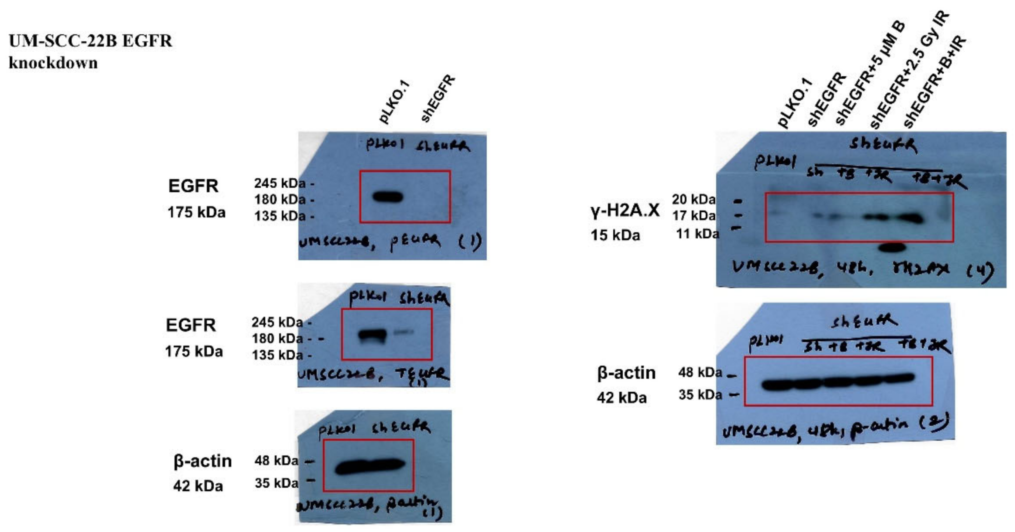

Figure S14: Original images for western blots for figure 5a and d (UM-SCC-22B EGFR knockdown).

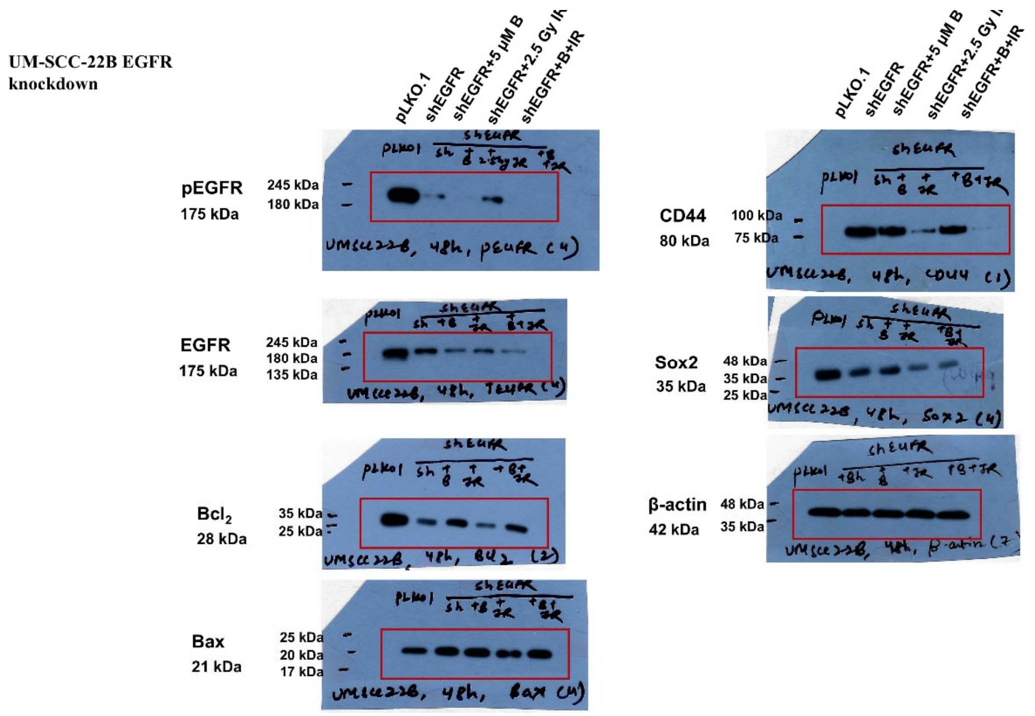

Figure S15: Original images for western blots for figure 5f (UM-SCC-22B EGFR knockdown).

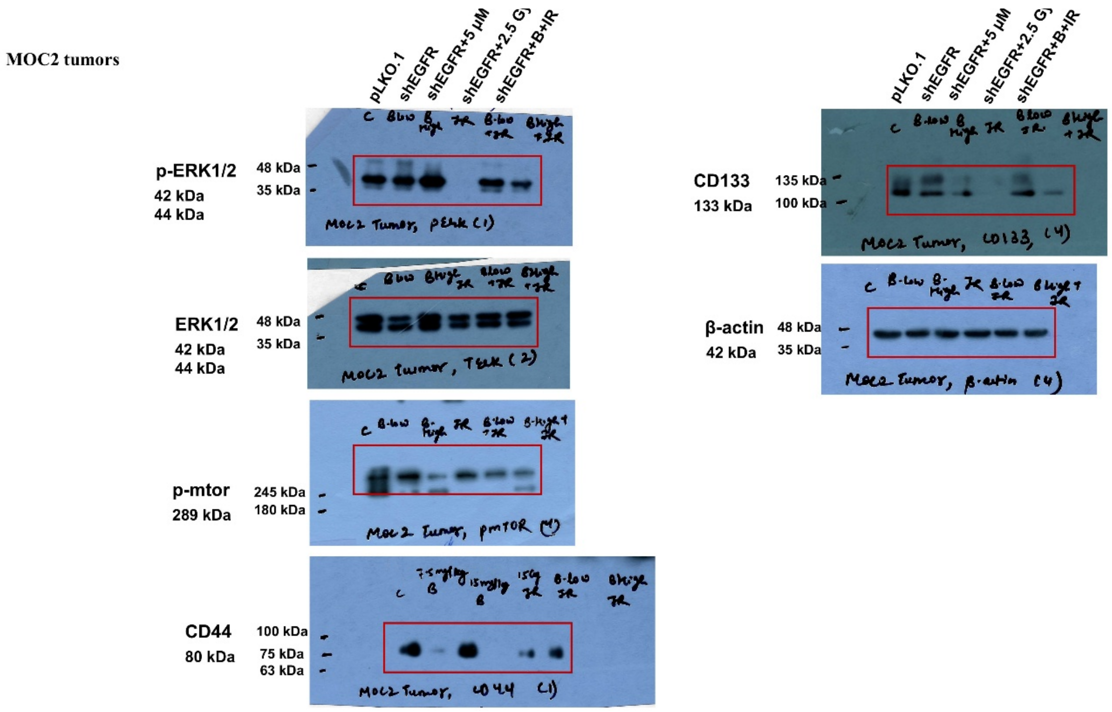

Figure S16: Original images for western blots for figure 7d (MOC2 tumors).

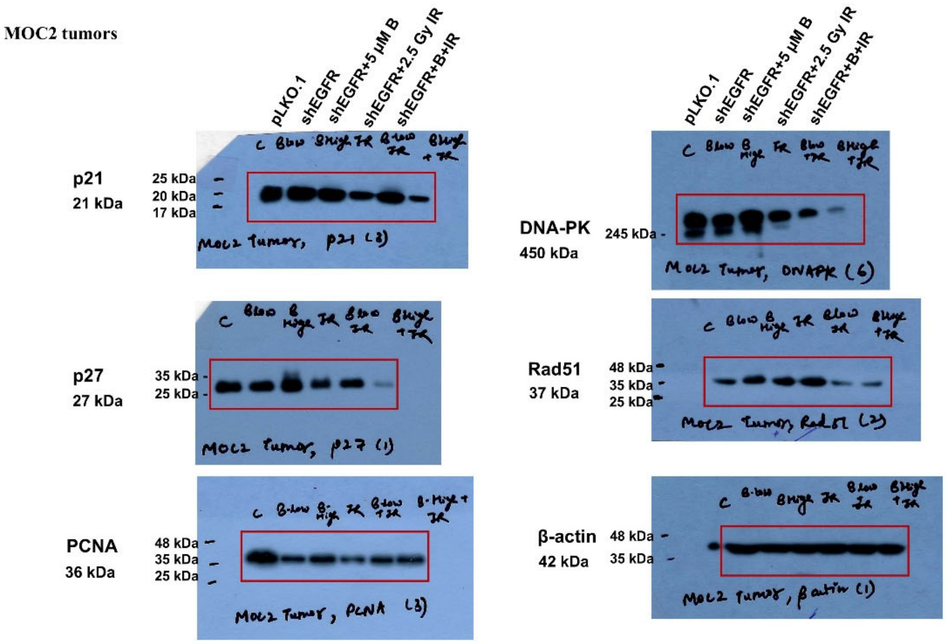

Figure S17: Original images for western blots for figure 7d (MOC2 tumors).
